# Applications of density functional theory to iron-containing molecules of bioinorganic interest

**DOI:** 10.3389/fchem.2014.00014

**Published:** 2014-04-29

**Authors:** Hajime Hirao, Nandun Thellamurege, Xi Zhang

**Affiliations:** Division of Chemistry and Biological Chemistry, School of Physical and Mathematical Sciences, Nanyang Technological UniversitySingapore, Singapore

**Keywords:** density functional theory, QMMM, iron-containing molecules, protein environment, enzyme reactions, catalysis

## Abstract

The past decades have seen an explosive growth in the application of density functional theory (DFT) methods to molecular systems that are of interest in a variety of scientific fields. Owing to its balanced accuracy and efficiency, DFT plays particularly useful roles in the theoretical investigation of large molecules. Even for biological molecules such as proteins, DFT finds application in the form of, e.g., hybrid quantum mechanics and molecular mechanics (QM/MM), in which DFT may be used as a QM method to describe a higher prioritized region in the system, while a MM force field may be used to describe remaining atoms. Iron-containing molecules are particularly important targets of DFT calculations. From the viewpoint of chemistry, this is mainly because iron is abundant on earth, iron plays powerful (and often enigmatic) roles in enzyme catalysis, and iron thus has the great potential for biomimetic catalysis of chemically difficult transformations. In this paper, we present a brief overview of several recent applications of DFT to iron-containing non-heme synthetic complexes, heme-type cytochrome P450 enzymes, and non-heme iron enzymes, all of which are of particular interest in the field of bioinorganic chemistry. Emphasis will be placed on our own work.

## Introduction

Density functional theory (DFT) has been playing increasingly important roles in many research activities of science and engineering in recent decades and has already become a mainstay for the quantum mechanical investigations of a broad range of complex molecular systems that are of interest in chemistry, biology, and physics (Parr and Yang, 1989; Kohn et al., 1996; Baerends and Gritsenko, 1997; Kohn, 1999; Koch and Holthausen, 2001; Zhao and Truhlar, 2008; Perdew et al., 2009; Burke, 2012; Cohen et al., 2012). DFT offers viable computational protocols with a good balance between accuracy and computational cost. This feature is particularly useful when one intends to investigate large molecular systems, to which the application of accurate *ab initio* methods may be difficult or even impossible. The availability of user-friendly software packages greatly assists in applying DFT calculations to individual specific problems.

In DFT, electronic energy *E* is expressed as a functional of electron density, viz.,

(1) $$\begin{array}{l} E=E\left[\rho (r)\right]\\ \;=T\left[\rho (r)\right]+V_{\text{ne}}\left[\rho (r)\right]+V_{\text{ee}}\left[\rho (r)\right]\text{​}, \end{array}$$

where *T* is the total kinetic energy of electrons, *V*_ne_ the potential energy resulting from an external potential and *V*_ee_ the electron–electron repulsion energy. Electron density ρ(**r**) for an *N*-electron system is defined as

(2) $$\rho (r)=N\int \cdots \int {\left|\Psi (x_{1},\;x_{2},\ldots ,x_{N})\right|}^{2}ds_{1}dx_{2}\ldots dx_{N},$$

where **x**_*i*_ collectively denote spatial (**r**_*i*_) and spin (*s*_*i*_) coordinates. In stark contrast to the wave function Ψ that depends on 3 × *N* (space) + *N* (spin) = 4*N* variables, ρ(**r**) contains only three spatial variables, implying that *E* may be obtained in a much more straightforward manner using ρ(**r**). In 1964, Hohenberg and Kohn proved that there is a one-to-one correspondence between the ground-state density and the external potential (Hohenberg and Kohn, 1964). They also showed that the variational principle holds for the ground-state energy. The variational principle and the Levy constrained-search formulation of DFT (Levy, 1979) ensure that *E* can be determined by minimizing it with respect to some *N*-representable trial electron densities.

Despite the fundamental importance of the Hohenberg–Kohn theorems, they do not provide explicit forms of the energy functionals in Equation 1 (except *V*_ne_). To proceed, one needs to know how *T* and *V*_ee_ are expressed as functionals of ρ(**r**). A practical approach to this problem was proposed by Kohn and Sham (1965). Their approach attempts to describe the real electron density by way of non-interacting electrons that are described using a Slater determinant of molecular orbitals (MOs) [or Kohn–Sham (KS) orbitals]. When the density is treated this way, the total energy is written as

(3) $$E[\rho (r)]=T_{\text{s}}[\rho (r)]+V_{\text{ne}}[\rho (r)]+J[\rho (r)]+E_{\text{XC}}[\rho (r)]$$

where *T*_s_[ρ(**r**)] is the kinetic energy of non-interacting electrons, *J*[ρ(**r**)] the classical electron-electron repulsion energy (Hartree energy), and *E*_XC_[ρ(**r**)] the exchange-correlation energy [= (*T* − *T*_s_) + (*V*_ee_ − *J*)]. The first term *T*_s_[ρ(**r**)], which accounts for a large portion of *T*[ρ(**r**)], may now be calculated using KS orbitals as in Hartree–Fock calculations.

Nevertheless the explicit form of *E*_XC_ remains unknown. In practice, KS equations are solved by employing an approximate *E*_XC_; as such, the accuracy of DFT energy depends critically on the quality of *E*_XC_. So far, a number of exchange-correlation functionals have been developed, by either constraint satisfaction or semi empirical fitting (Perdew et al., 2005). *E*_XC_ functionals are classified into the following five major levels (rungs) of “Jacob's Ladder” according to their types: namely, local spin density approximation (LSDA), generalized gradient approximation (GGA), meta-GGA, hyper-GGA, and generalized random phase approximation (Perdew and Schmidt, 2001). In the field of chemistry, Gaussian- or Slater-type atomic orbitals (AOs) are usually used as basis sets of KS orbitals, while GGA, meta-GGA, or hyper-GGA functionals are mainly used to approximate *E*_XC_. When KS equations are solved for periodic solids or nanomaterials, plane-wave basis sets and pseudopotentials are used often with LSDA or GGA functionals.

When AO-type localized basis sets are used, the maximum number of atoms that DFT can manage is normally around a few hundred. Nevertheless, DFT is useful in investigating even larger systems such as metalloenzymes that contain thousands of atoms or more, even though the application of DFT to an entire enzyme may be prohibitive. Two of the more practical approaches are (1) to extract relatively important active site atoms and then apply DFT to this active site model, and (2) to divide the system into a few layers first, and then apply different computational methods to individual layers in a hybrid manner to describe the entire system effectively. The latter hybrid calculation can be performed, e.g., by using a QM/MM method (Warshel and Levitt, 1976; Field et al., 1990; Maseras and Morokuma, 1995; Gao, 1996; Humbel et al., 1996; Svensson et al., 1996; Murphy et al., 2000; Cui et al., 2001; Friesner and Guallar, 2005; Nam et al., 2005; Riccardi et al., 2006; Vreven et al., 2006; Senn and Thiel, 2007a,b, 2009; Lin and Truhlar, 2007; Hu and Yang, 2008, 2009; Kamerlin et al., 2009; Chung et al., 2011a; Hirao and Morokuma, 2011a; Sameera and Maseras, 2012) There are three major schemes of QM/MM, i.e., mechanical-embedding (ME), electronic-embedding (EE), and polarizable-embedding (PE) schemes, which differ in how the electrostatic interaction between QM atoms and remaining atoms is treated (Senn and Thiel, 2007a,b, 2009). In ME-QM/MM, the electrostatic interaction is evaluated as the interaction between the gas-phase state of QM atoms and the point charges of MM atoms. Thus, the polarization effect of the QM electron density caused by MM point charges is not taken into account. The EE-QM/MM method can describe such QM polarization. PE-QM/MM further allows the MM atoms to be polarized, and thus the effect of mutual polarization between QM atoms and surrounding atoms can be taken into account. Many conventional QM/MM methods evaluate the total energy of the entire system in an additive manner. That is, the energy *E*_QM/MM_ is given as

(4) $$E_{\text{QM/MM}}=E_{\text{QM}}+E_{\text{MM}}+E_{\text{QM}-\text{MM}}.$$

By contrast, the ONIOM (our own N-layer integrated MO molecular mechanics) method allows one to combine QM and MM (QM:MM) in a subtractive (extrapolative) fashion (Maseras and Morokuma, 1995), in which case the total energy is

(5) $$E_{\text{QM/MM}}=E_{\text{MM}}^{\text{R}}+E_{\text{QM}}^{\text{M}}-E_{\text{MM}}^{\text{M}}$$

where superscripts R and M denote real (large) and model (small) systems, respectively. It should be noted that the “MM region” in an additive QM/MM means the atoms outside the QM region. However, in a subtractive QM/MM, MM is applied to both the entire system and the small model system (i.e., “QM region”). Thus, even though both methods give more or less similar approximations to the total energy, the meanings of the individual subsystems, especially the subsystems to which MM is applied, could be somewhat different. In the subtractive ONIOM approach, two (or more) different levels of QM methods can also be naturally combined [i.e., ONIOM(QM:QM')], and such hybrid QM/QM methods are useful, e.g., in exploring the mechanisms of chemical reactions catalyzed by bulky transition-metal catalysts within a reasonable computational time.

With these theoretical frameworks and tools in hand, it is obvious that there could be an infinite number of potential research targets to be explored by DFT calculations. However, in this paper, we shall focus on Fe/O-containing molecules, or more specifically, synthetic non-heme iron complexes (Costas et al., 2000; Nam, 2007; Que, 2007; Que and Tolman, 2008; McDonald and Que, 2013; Nam et al., 2014), heme-type cytochrome P450 enzymes (Dawson and Sono, 1987; Sono et al., 1996; Guengerich, 2001, 2008; Denisov et al., 2005; Ortiz de Montellano, 2005, 2010; Sligar et al., 2005; Makris et al., 2006; Groves, 2006; Isin and Guengerich, 2007; Poulos, 2014), and non-heme iron enzymes (Feig and Lippard, 1994; Que and Ho, 1996; Wallar and Lipscomb, 1996; Hegg and Que, 1997; Lange and Que, 1998; Solomon et al., 2000, 2009; Merkx et al., 2001; Baik et al., 2003; Stubbe et al., 2003; Costas et al., 2004; Tshuva and Lippard, 2004; Abu-Omar et al., 2005; Bollinger and Krebs, 2006, 2007; Neidig et al., 2006; Krebs et al., 2007; Friedle et al., 2010; van der Donk et al., 2010).

Our motivation to study these iron-containing molecules comes from the remarkable catalytic machinery operating in iron enzymes. In fact, iron enzymes display diverse reactivity patterns, e.g., as oxygenases [monooxygenases (Equation 6) or dioxygenases (Equation 7)] or as peroxidases (Dawson and Sono, 1987; Wang et al., 2013a; Poulos, 2014).

(6) $$\text{XH + }O_{2}+{\text{AH}}_{2}\to \text{X}\left(O\right)\text{H + }{\text{H}}_{2}O\text{ + A}$$

(7) $$\text{XH + }O_{2}\to \text{X}\left(O_{2}\right)\text{H}$$

However, the active intermediates of iron enzymes, such as compound I of P450, (Dawson and Sono, 1987; Sono et al., 1996; Schlichting et al., 2000; Davydov et al., 2001; Guengerich, 2001, 2008; Denisov et al., 2005; Ortiz de Montellano, 2005, 2010; Sligar et al., 2005; Groves, 2006; Makris et al., 2006; Isin and Guengerich, 2007; Rittle and Green, 2010; Poulos, 2014) intermediate J of taurine dioxygenase (TauD) (Krebs et al., 2007), intermediate Q of soluble methane monooxygenase (sMMO) (Shu et al., 1997; Solomon et al., 2000; Merkx et al., 2001; Baik et al., 2003; Bollinger and Krebs, 2006), and intermediate X of ribonucleotide reductase (RNR) (Solomon et al., 2000; Bollinger and Krebs, 2006), are often difficult to trap and spectroscopically characterize in normal turnover conditions. Theoretical DFT and DFT/MM calculations have useful roles to play in providing insights into the elusive aspects of iron enzymes such as the nature of short-lived intermediates and chemical reaction mechanisms.

## Applications of DFT

### Synthetic iron complexes

#### Reactivity patterns

Transition metals in enzymes or synthetic complexes enable otherwise difficult chemical transformations such as C–H activation (Groves et al., 1978; Ryabov, 1990; Shilov and Shul'pin, 1997; Shilov and Shteinman, 1999; Jia et al., 2001; Labinger and Bercaw, 2002; Lehnert et al., 2002; Yoshizawa, 2002; Kakiuchi and Chatani, 2003; Goldberg and Goldman, 2004; Godula and Sames, 2006; Groves, 2006, 2014; Siegbahn and Borowski, 2006; Bergman, 2007; Chen and White, 2007; Seregin and Gevorgyan, 2007; Davies and Manning, 2008; Balcells et al., 2010; Lyons and Sanford, 2010; Ackermann, 2011; Yamaguchi et al., 2012; Webb et al., 2013; Yosca et al., 2013). The activation or functionalization of C–H bonds of cheap and abundant substrates such as alkanes is one of the most important goals in transition-metal catalysis.

**Scheme 1:**
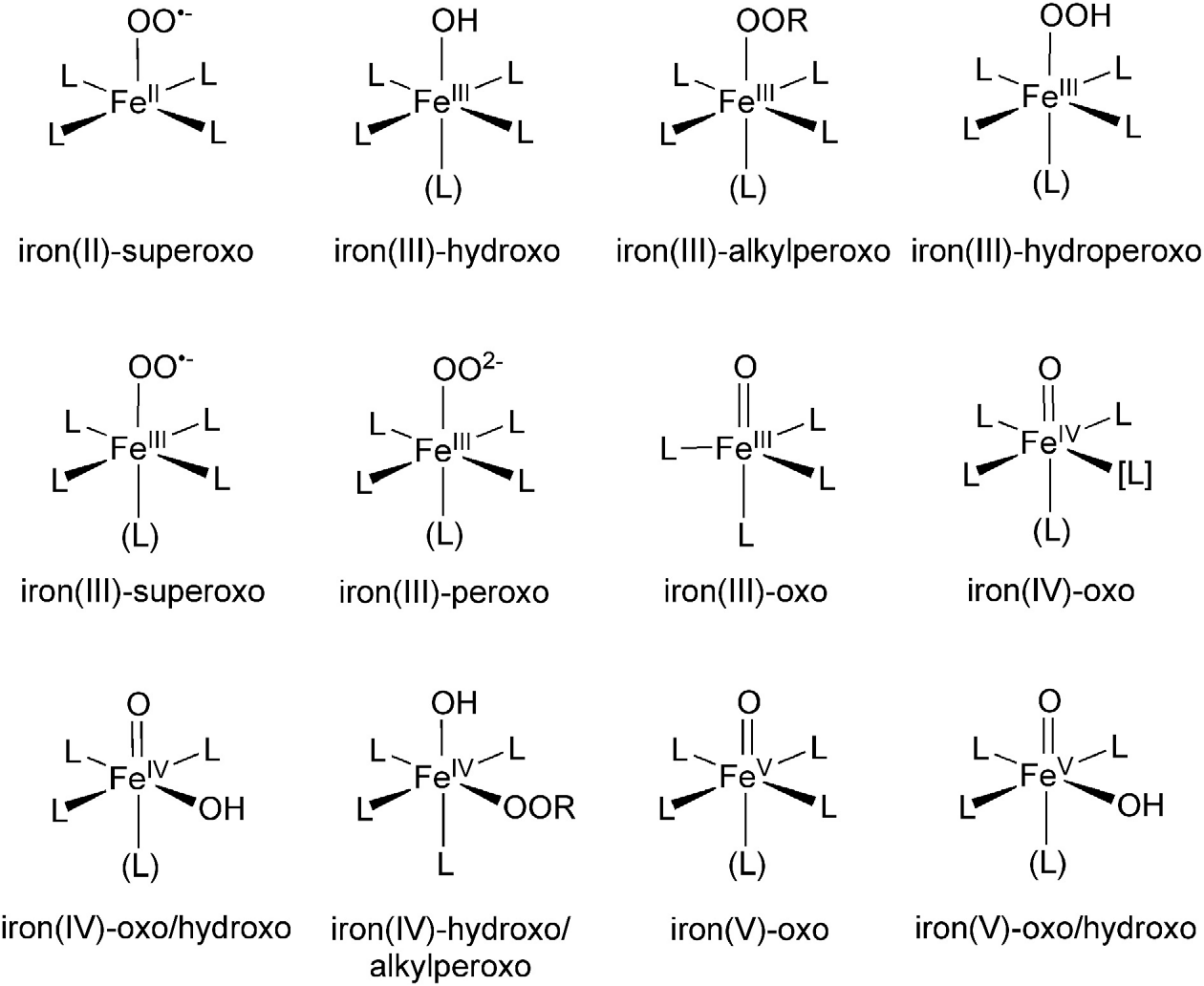
**Some of the (oxygen-bound) non-heme iron complexes studied by DFT calculations.** L denotes atoms of non-oxygen ligands, but the use of the common symbol does not necessarily mean that the ligand atoms are equivalent.

Iron-containing cytochrome P450 enzymes (P450s) are capable of activating C–H bonds (Groves, 1985), and thus the development of synthetic, biomimetic analogs that resemble P450s has been actively pursued (Lim et al., 2003; Rohde et al., 2003; Kaizer et al., 2004; Bukowski et al., 2005; Kim et al., 2005; Sastri et al., 2005; Bautz et al., 2006; Thibon et al., 2008; Yoon et al., 2009; Lee et al., 2010; Li et al., 2010, 2011; Cho et al., 2011a; Hohenberger et al., 2012). Theoretical studies of such biomimetic models may not only identify the key elements that determine their chemical reactivities, but may also provide insight into intermediates and reactivities of parent enzymes (Shaik et al., 2007a; de Visser et al., 2013). To date, DFT calculations have been applied extensively to various types of non-heme iron species (Scheme 1) (Bassan et al., 2002, 2005a,b; Roelfes et al., 2003; Decker and Solomon, 2005; Kumar et al., 2005; Quinonero et al., 2005; Berry et al., 2006; Bernasconi et al., 2007, 2011; de Visser, 2006, 2010; Hirao et al., 2006a, 2008a,b, 2011; Rohde et al., 2006; Decker et al., 2007; de Visser et al., 2007, 2011; Johansson et al., 2007; Noack and Siegbahn, 2007; Sastri et al., 2007; Sicking et al., 2007; Bernasconi and Baerends, 2008, 2013; Comba et al., 2008; Dhuri et al., 2008; Fiedler and Que, 2009; Klinker et al., 2009; Wang et al., 2009a, 2013b; Cho et al., 2010, 2012a, 2013; Geng et al., 2010; Chen et al., 2011; Chung et al., 2011b; Seo et al., 2011; Shaik et al., 2011; Vardhaman et al., 2011; Wong et al., 2011; Ye and Neese, 2011; Gonzalez-Ovalle et al., 2012; Gopakumar et al., 2012; Latifi et al., 2012; Mas-Ballesté et al., 2012; McDonald et al., 2012; Van Heuvelen et al., 2012; Ansari et al., 2013; Kim et al., 2013; Lee et al., 2013; Sahu et al., 2013; Tang et al., 2013; Ye et al., 2013; Hong et al., 2014; Sun et al., 2014). The intriguing reactivity patterns of these complexes are the result of active involvement of electrons in d-type MOs, which gives rise to multi-state scenarios (Shaik et al., 1998; Schröder et al., 2000; Schwarz, 2011).

**Figure 1:**
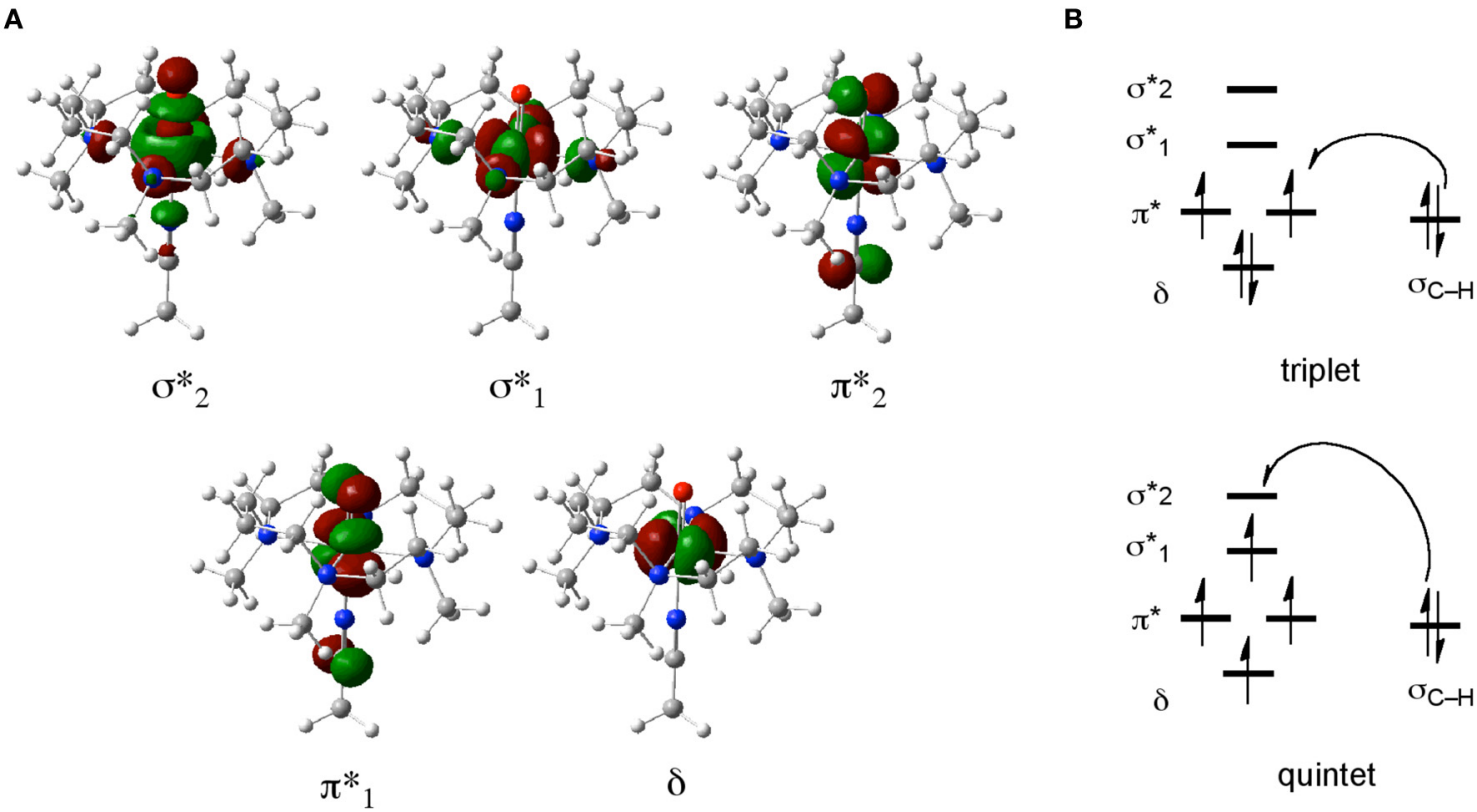
**(A)** Five key d-type MOs of the TMC iron(IV)-oxo complex. **(B)** Typical electron-shift patterns for the reactions of non-heme iron(IV)-oxo complexes.

When we study the chemical reactions of these non-heme iron complexes using DFT, a main goal is to obtain structural and energetic information for various transition states and intermediates on reaction pathways. In addition, by analyzing KS orbitals or some sort of transformed localized orbitals, one can gain valuable chemical insight into the electronic reorganization during the reactions. Of all the species in Scheme 1, iron(IV)-oxo complexes are particularly important in view of their resemblance to iron(IV)-oxo porphyrin π-cation radical species (compound I or Cpd I) of P450s. Figure 1A depicts the five important d-type orbitals of a non-heme iron(IV)-oxo complex supported by a 1,4,8,11-tetramethyl-1,4,8,11-tetraazacyclotetradecane (TMC) ligand and an acetonitrile axial ligand. Previous DFT studies identified typical electron-shift patterns for the reactions of non-heme iron(IV)-oxo complexes (Figure 1B) (Hirao et al., 2006a; Shaik et al., 2007a). In the lower-spin triplet state, the δ orbital is doubly occupied and each of the two π^*^ orbitals is singly occupied in the initial stage. In the C–H bond activation step, an electron migrates from the substrate to a π^*^ orbital. As a result, the formal oxidation state of iron becomes +3, with one of the two π^*^ orbitals being singly occupied. By contrast, in the reaction of a higher-spin quintet state, an electron tends to transfer from the substrate C–H σ bond (σ_C−H_) to the vacant σ^*^_z2_ orbital (denoted σ^*^_2_ in Figure 1), resulting again in a +3 Fe oxidation state. In this case, however, there is an increase in the number of unpaired electrons on the iron center. This gives rise to an enhanced exchange stabilization of the system, which results in a substantially lower energy barrier in the quintet state. Because the σ^*^_2_ orbital extends along the Fe–O axis, the charge transfer in the quintet state requires that the substrate should approach the iron(IV)-oxo moiety from just above the oxo unit to achieve the maximum overlap between σ_C−H_ and σ^*^_2_. Consequently, the transition state tends to have a markedly upright geometry, with the C, H, O, and Fe atoms aligned in a collinear configuration.

#### Reactions involving O–O bond cleavage

**Scheme 2:**
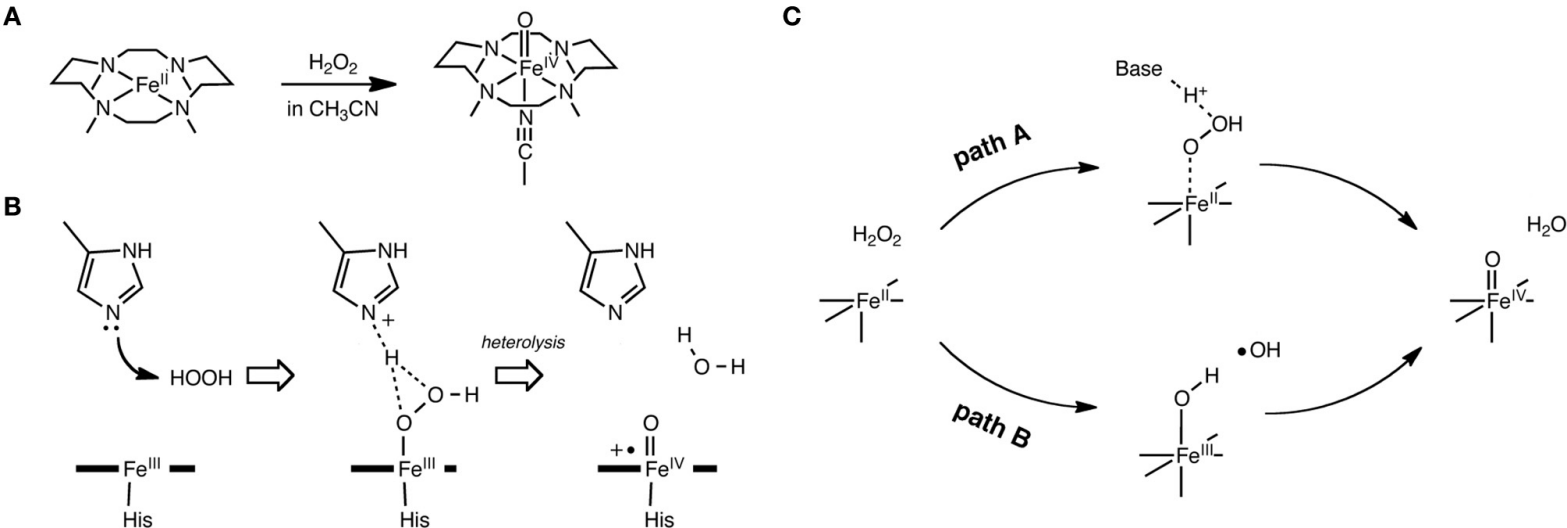
**(A)** Iron(IV)-oxo formation from TMC Fe(II) and H_2_O_2_. **(B)** Cpd I formation in heme peroxidases. **(C)** The two pathways examined. Adapted from Hirao et al. (2011) with permission from the American Chemical Society.

**Scheme 3:**
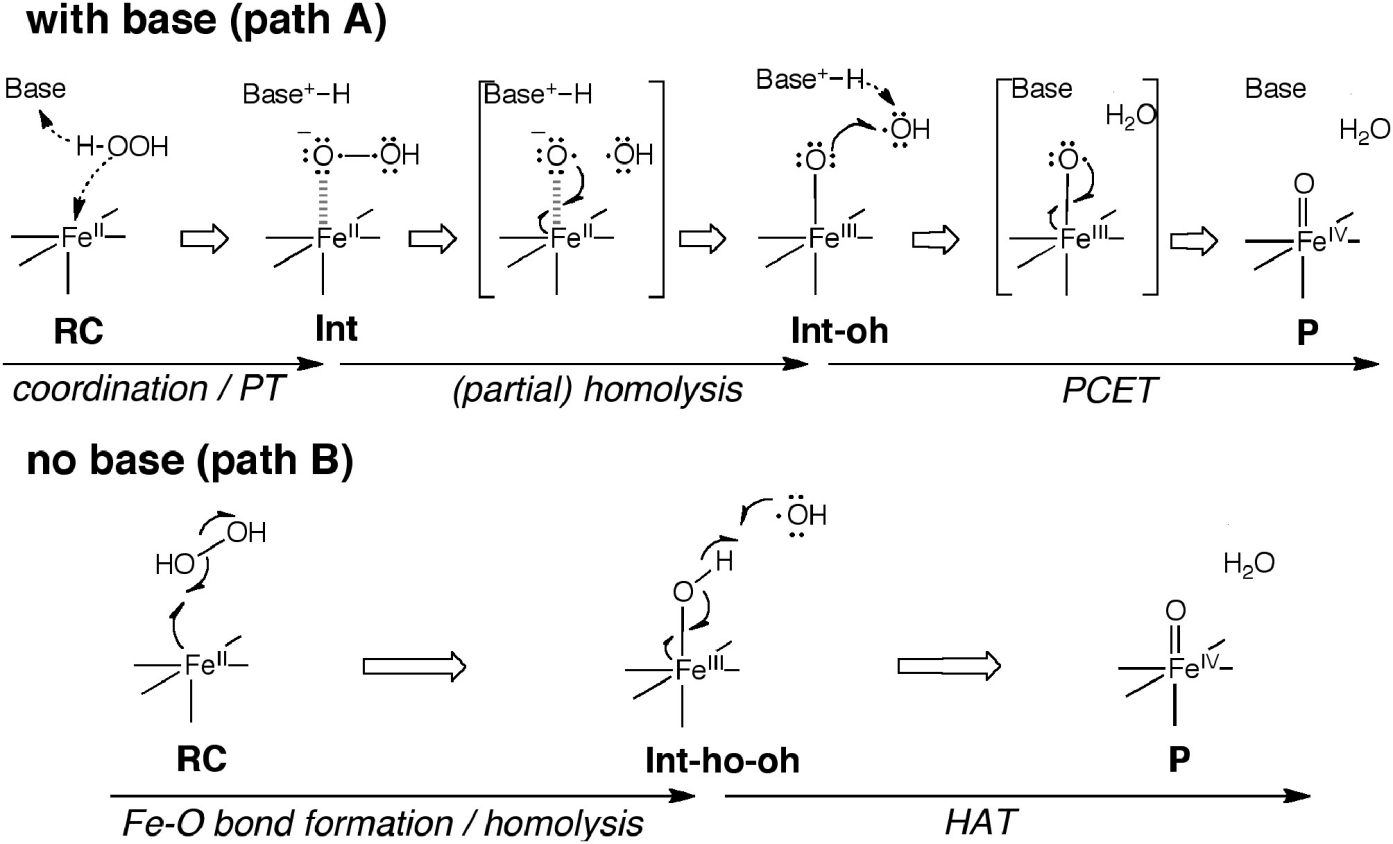
**Schematic illustration of electron reorganization during the reactions in Scheme 2C.** Adapted from Hirao et al. (2011) with permission from the American Chemical Society.

**Figure 2:**
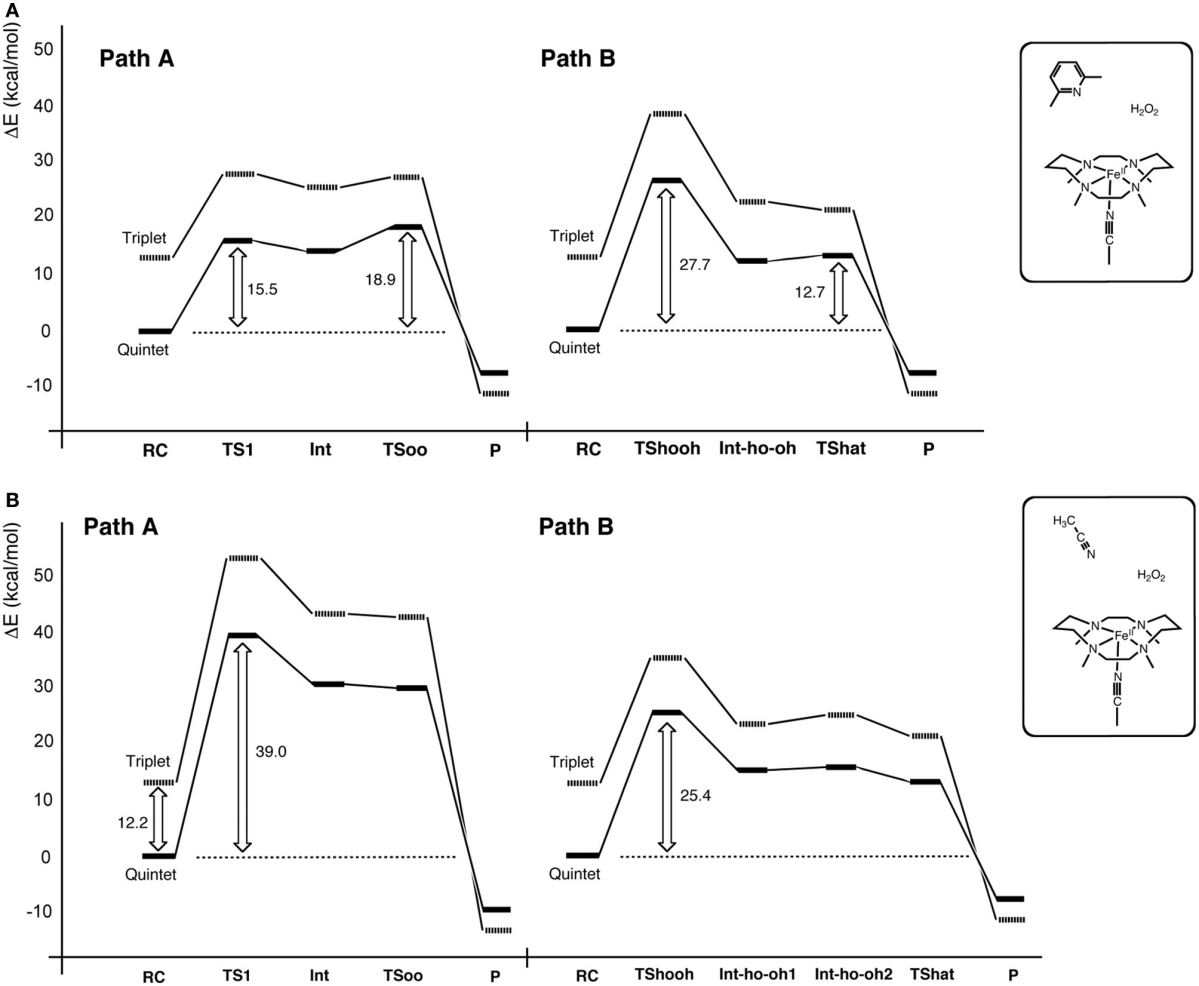
**Energy profiles for the reactions in Scheme 2C in the presence (A) and absence (B) of 2,6-lutidine.** Adapted from Hirao et al. (2011) with permission from the American Chemical Society.

Another interesting conundrum regarding synthetic iron(IV)-oxo species is how an iron(IV)-oxo species is itself produced from other species. In P450s, Cpd I is formed in the catalytic cycle when the O–O bond of the precursor intermediate, Cpd 0, undergoes heterolytic cleavage (Dawson and Sono, 1987; Sono et al., 1996; Guengerich, 2001, 2008; Denisov et al., 2005; Ortiz de Montellano, 2005, 2010; Sligar et al., 2005; Makris et al., 2006; Groves, 2006; Isin and Guengerich, 2007; Poulos, 2014). The O–O bond cleavage also constitutes a critical step in ferryl formation in non-heme enzymes (Krebs et al., 2007). Rohde et al. showed experimentally, that a synthetic TMC iron(IV)-oxo is formed at −40°C with 3 equivalents of H_2_O_2_ in acetonitrile (Rohde et al., 2003). More recently, Li et al. reported that a TMC iron(IV)-oxo species can be generated by reacting a TMC Fe(II) complex with a stoichiometric amount of H_2_O_2_ in acetonitrile in the presence of 2,6-lutidine (Scheme 2A) (Li et al., 2010). Interestingly, the addition of 2,6-lutidine accelerated the reaction rate and enhanced the reaction yield. It was postulated that the role of 2,6-lutidine is analogous to that played by a distal histidine in the formation of the Cpd I intermediate in heme peroxidases (Scheme 2B) (Poulos and Kraut, 1980; Newmyer and Ortiz de Montellano, 1995; Tanaka et al., 1997; Wirstam et al., 1999; Derat and Shaik, 2006; Chen et al., 2008; Poulos, 2014). The DFT calculations performed by Hirao et al. support this hypothesis (Path A in Scheme 2C) and provide additional insights into electronic details of the reaction mechanism (Scheme 3 and Figure 2A) (Hirao et al., 2011). If there is no base in the system, an acetonitrile molecule in the solvent could act as an acid-base catalyst in Path A. However, the calculated energy barrier for this acetonitrile-catalyzed reaction (39 kcal/mol) is too high for this reaction to proceed (Figure 2B). This is not in accordance with the fact that an iron(IV)-oxo can be produced even in the absence of 2,6-lutidine. In an attempt to find an alternative pathway, Hirao et al. examined Path B (Schemes 2C, 3), which is initiated by a homolytic O–O bond cleavage of H_2_O_2_. The energy barrier for this reaction was much lower (25.4 kcal/mol, see Figure 2B). In light of these results, they suggested that in the absence of 2,6-lutidine, the reaction follows Path B to afford the same iron(IV)-oxo product.

As seen above, the mode of O–O bond cleavage in the reactions of synthetic non-heme complexes is a mechanistically intriguing aspect. In the above case, the mode was either (virtually) heterolytic or homolytic, depending on the presence of an acid-base catalyst. More specifically, these processes can be viewed essentially as O–O cleavage that gives OH^−^ or OH•. Another type of O–O bond cleavage has been reported recently. Kim et al. found that a non-heme high-spin iron(III)-hydroperoxo species bearing a macrocyclic TMC ligand is capable of mediating sulfoxidation of thioanisole (Kim et al., 2013). Detailed DFT calculations and orbital analyses characterized the O–O bond cleavage as heterolytic; however, in this case, the high-spin Fe(III)OOH^2+^ species heterolytically splits into Fe(III)O^+^ and OH^+^. When the OH^+^ forms a bond with thioanisole, the proton of OH^+^ is donated back to the Fe(III)O^+^ moiety. All these events take place in a concerted fashion, and hence, an OH^+^ ion is not produced as an intermediate.

#### Ligand effects

**Scheme 4:**
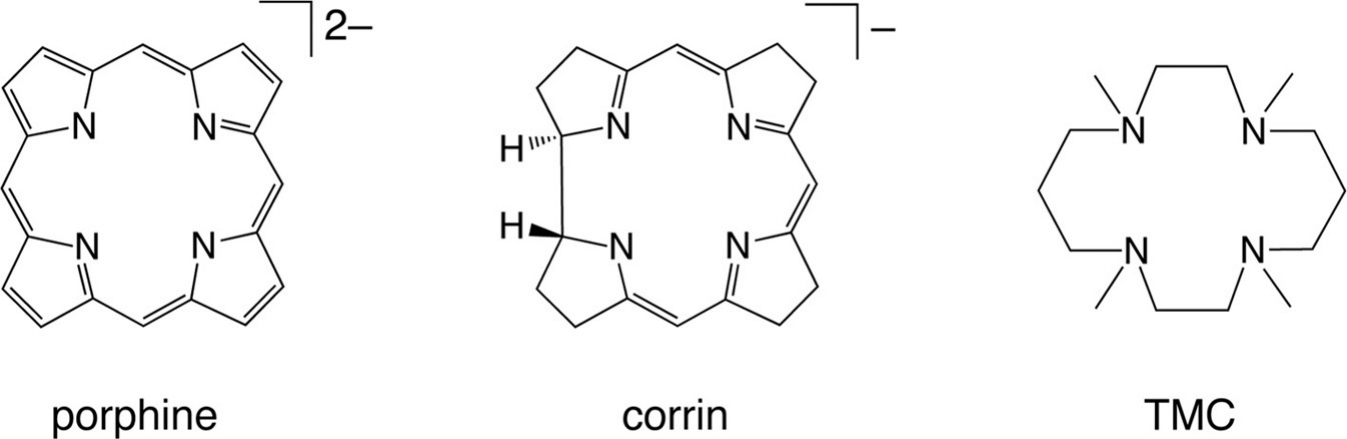
**The three ligands studied by Myradalyyev et al.** Reprinted from Myradalyyev et al. (2013) with permission from Elsevier.

**Figure 3:**
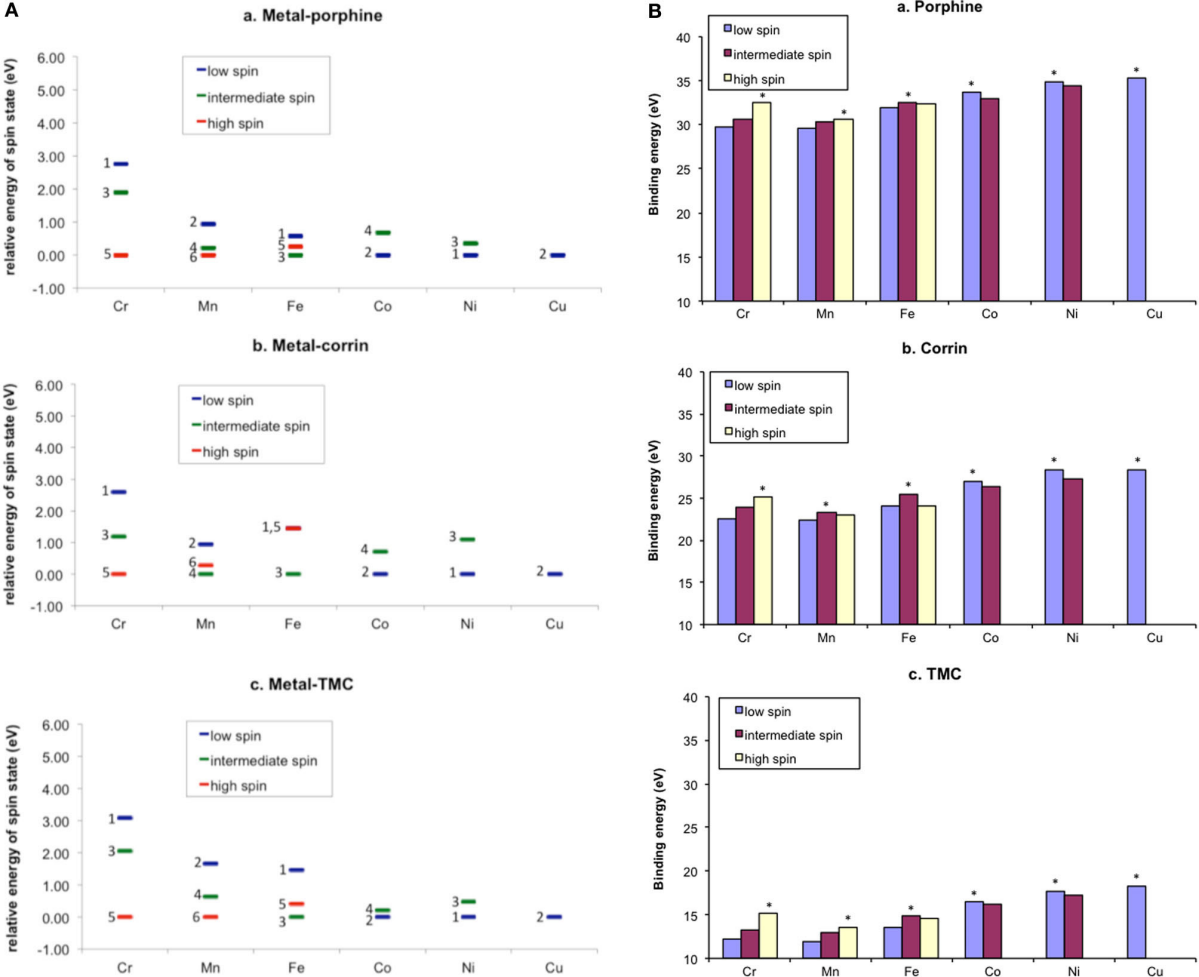
**(A)** Relative stability of various complexes metal(II)-ligand complexes in different spin states. **(B)** Binding energy of the complexes. Adapted from Myradalyyev et al. (2013) with permission from Elsevier.

The reactivity and many other properties of the iron (or more broadly, metal) center in enzymes and synthetic complexes are affected significantly by coordinating ligands. Therefore, the theoretical evaluation of ligand effects is an important task of computational chemistry. Myradalyyev et al. performed a comparative B3LYP DFT study of the interactions between the porphine, corrin, and TMC ligands (Scheme 4) and several metal ions (Cr^2+^, Mn^2+^, Fe^2+^, Co^2+^, Ni^2+^, and Cu^2+^) (Myradalyyev et al., 2013). The computationally determined ground spin states were in good agreement with the available experimental data (Figure 3A). The binding affinity was shown to increase in the order Mn^2+^ < Cr^2+^ ~ Fe^2+^ < Co^2+^ < Ni^2+^ < Cu^2+^ in all ligands (Figure 3B). The relative binding strength is determined by several factors, such as the total charge of the ligand, spin promotion in the complexation, electrostatic/Pauli interactions, and ligand-to-metal or metal-to-ligand charge transfer (Feixas et al., 2009).

### Cytochrome P450 enzymes

#### Reactive species of P450s

DFT and DFT/MM studies have also played vital roles in advancing our understanding of the electronic structure and reactivity of P450s. It is commonly accepted that Cpd I acts as the reactive species of P450-dependent oxidation reactions, and several previous experiments support this idea (Schlichting et al., 2000; Davydov et al., 2001; Rittle and Green, 2010). The electronic structure of Cpd I and the electron reorganization patterns of the substrate oxidation reactions of Cpd I have been discussed for several different spin states (Ogliaro et al., 2000a,b; Yoshizawa et al., 2001; de Visser et al., 2002; Himo and Siegbahn, 2003; Kamachi and Yoshizawa, 2003; Meunier et al., 2004; Hackett et al., 2005, 2007; Hirao et al., 2005; Shaik et al., 2005, 2007a,b, 2010a; Isobe et al., 2008, 2011, 2012; Shoji et al., 2008; Yamaguchi et al., 2009; Li et al., 2012; Rydberg et al., 2014). Computational studies to assess the reactivity of Cpd 0 have also been conducted (Ogliaro et al., 2002; Kamachi et al., 2003; Bach and Dmitrenko, 2006; Derat et al., 2006; Hirao et al., 2006b; Li et al., 2007). Overall, DFT calculations predicted that Cpd 0 is less reactive than Cpd I. Efforts to improve our understanding of the nature of P450 reactive species are still ongoing. Recently, Wang et al. examined sulfoxidation mediated by the Fe(III)(HOOH) complex of a P450 using DFT, and showed that the energy barrier is 5.3 kcal/mol, compared with 4.7 kcal/mol in the Cpd I reaction and 23.6 kcal/mol in the Cpd 0 reaction (Wang et al., 2013c). From these DFT results, they suggested that the Fe(III)(HOOH) complex could be an alternative reactive species for sulfoxidation.

#### Mechanism-based inactivation

The reactivity patterns of P450s are mechanistically interesting conundrums. P450s are also important enzymes in a practical sense, especially in the context of drug metabolism. The essential functions of P450s in Phase I drug metabolism are now well appreciated. An important feature of P450s in drug metabolism is their broad substrate specificity. Although >18,000 sequences of P450s have been identified thus far, humans have only 57, and drug metabolism is performed by only a few of these (Pelkonen et al., 2008; Guengerich, 2013; Poulos, 2014). As such, the inhibition of P450s through drug–drug interactions (DDIs) could adversely affect many metabolic reactions in the body.

DFT has begun to find applications in such practical issues of P450s. In particular, DFT can make significant contributions to the understanding of a special type of DDI, i.e., mechanism-based inactivation (MBI) (Zhou et al., 2005; Orr et al., 2012). There are two different types of MBI: quasi-irreversible and irreversible. In MBI, an inhibitor molecule first reacts with Cpd I to be converted to a metabolic intermediate (MI), and then the MI binds to the active site of a P450 quasi-irreversibly or irreversibly, resulting in the inhibition of the enzyme. In a quasi-irreversible MBI, the MI forms a coordination bond with the heme iron, whereas in an irreversible MBI, the MI forms a strong covalent bond with an amino acid residue or the porphyrin ligand. In either case, MBI involves a chemical reaction, which cannot be described by conventional docking simulations. Rather, one must use quantum mechanical methods such as DFT. In recent years, several research groups have reported DFT studies of P450 MBI (de Visser et al., 2004; Rydberg and Olsen, 2011; Hirao et al., 2012, 2013a,b; Taxak et al., 2012, 2013a,b).

**Figure 4:**
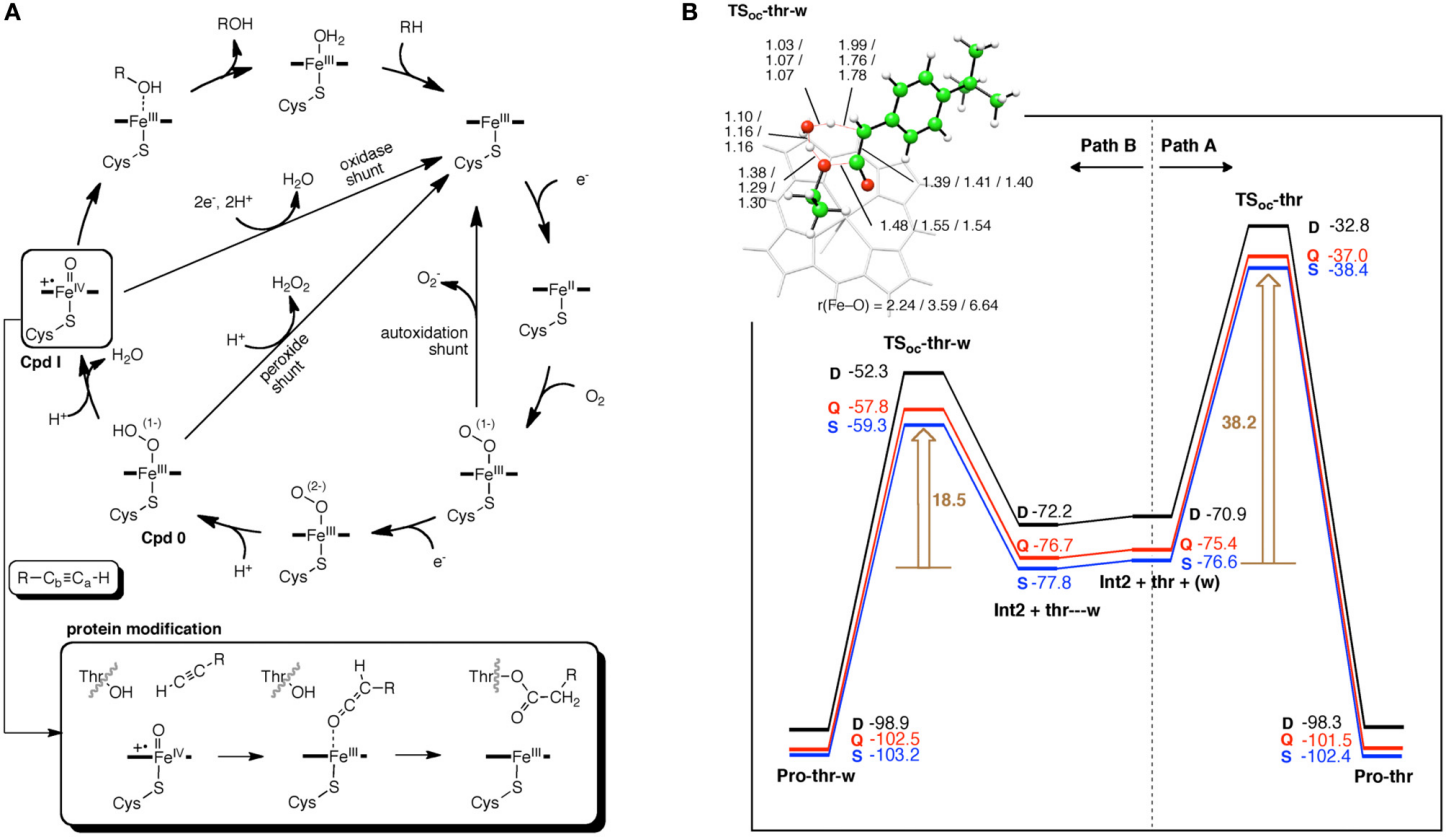
**(A)** Catalytic cycle of P450 (top) and a proposed mechanism of MBI caused by terminal acetylenes (bottom) **(B)** Energy diagrams (in kcal/mol) for the reactions of a ketene intermediate in the absence **(A)** and presence **(B)** of a water molecule. Adapted from Hirao et al. (2012) with permission from the American Chemical Society.

Hirao et al. studied the irreversible MBI caused by terminal acetylenes (Figure 4) (Hirao et al., 2012). Their calculations showed that without the involvement of a water molecule, covalent bond formation between the ketene-type metabolic intermediate and the catalytically essential threonine residue is difficult, because the corresponding energy barrier is too high (>38 kcal/mol). However, when a water molecule was allowed to participate in the reaction, the barrier was reduced by about 20 kcal/mol. This result suggested that a water molecule plays a crucial role in “terminating” the enzymatic function of P450.

**Scheme 5:**
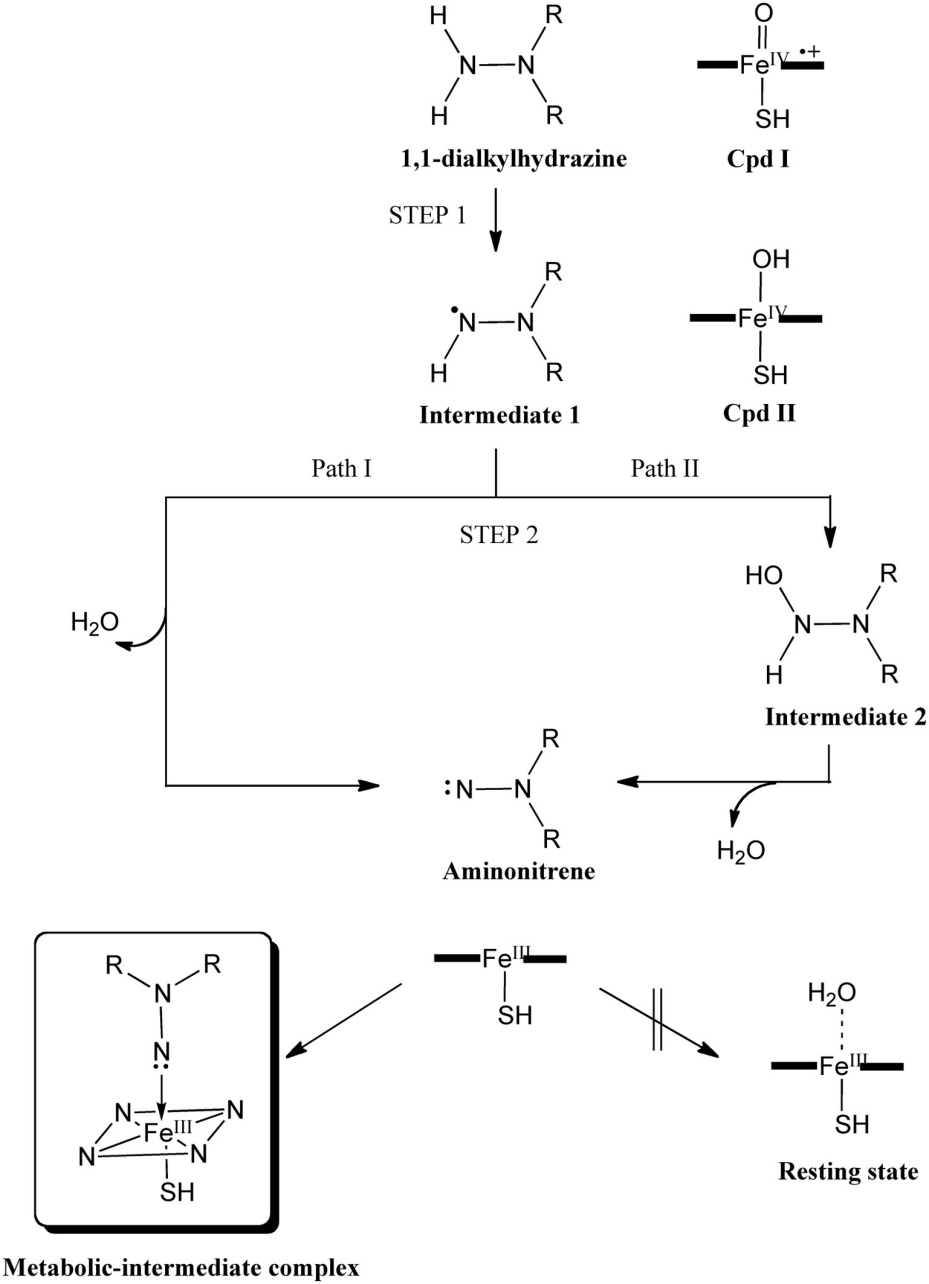
**Proposed mechanism of the MBI of P450 by UDMH.** Adapted from Hirao et al. (2013a) with permission from Wiley-VCH.

**Figure 5:**
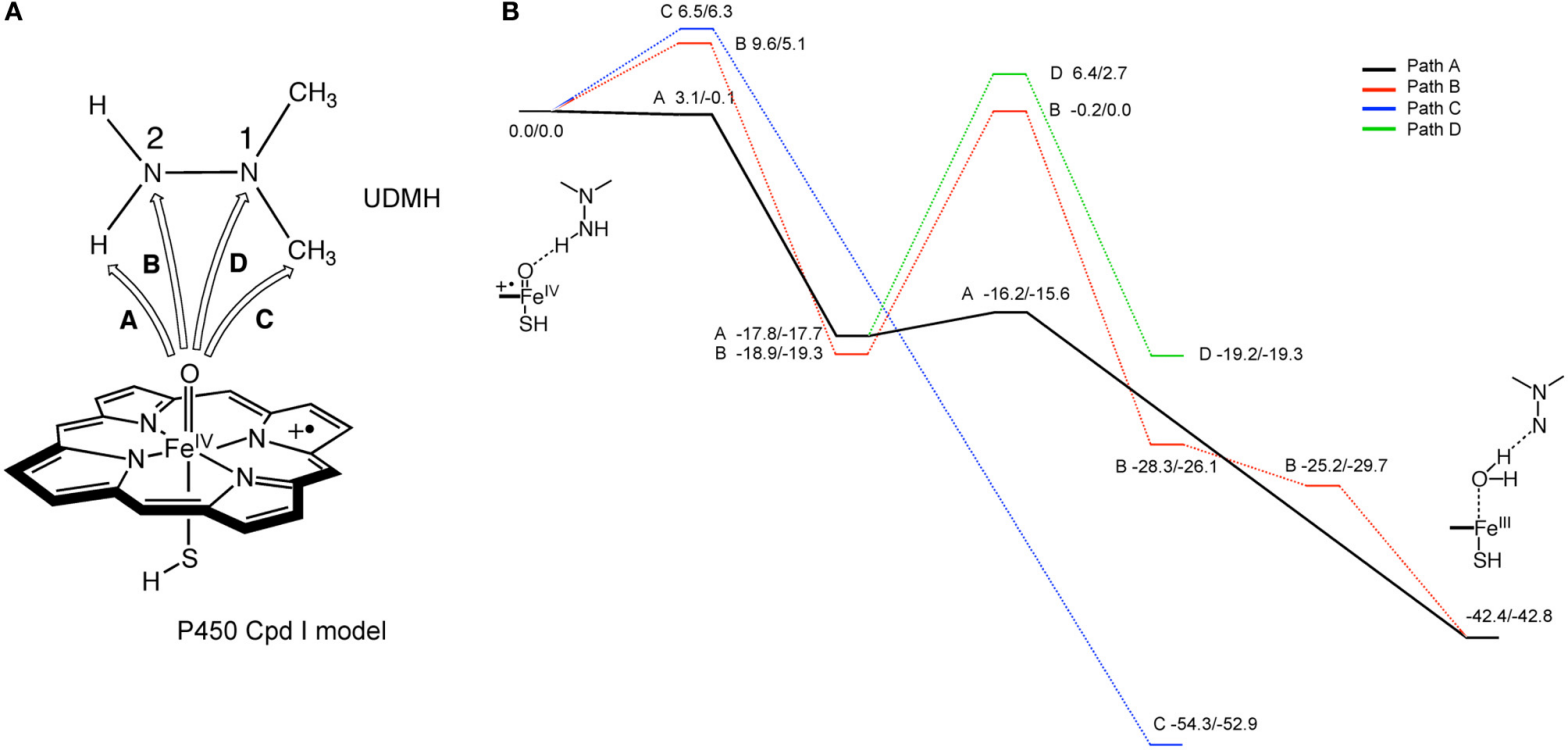
**(A)** Reaction pathways considered for the MI formation from dimethylhydrazine. **(B)** Energy profiles (kcal/mol) for the four pathways considered. Adapted from Hirao et al. (2013a) with permission from the Wiley-VCH.

Hirao et al. also studied the mechanism of metabolic-intermediate formation in the quasi-irreversible MBI of P450 caused by 1,1-dialkylhydrazine (or unsymmetrical dimethylhydrazine, UDMH) (Hirao et al., 2013a). For this MBI, hydrazine is converted to an aminonitrene metabolic intermediate, which in turn binds to the heme iron. However, there are at least two possible mechanisms for this reaction (Scheme 5) (Ortiz de Montellano, 2005). In the first mechanism, two hydrogen atoms are successively abstracted from the terminal nitrogen by Cpd I or another ferryl species, Cpd II (Green et al., 2004). In the other mechanism, the first H-abstraction is followed by N-oxidation. Other mechanisms may also be possible in which the first step is not H-abstraction from a N–H bond. Hirao et al. examined four different pathways using DFT as shown in Figure 5A, and found that the preferred reaction involves H-abstraction from the N–H bond in the first step. After the H-abstraction, the substrate radical undergoes another H-abstraction from the same nitrogen, to yield an aminonitrene species. The DFT study also showed that the aminonitrene MI coordinates to the heme in a somewhat tilted orientation, and that aminonitrene binds to the heme more strongly than a water molecule.

**Scheme 6:**
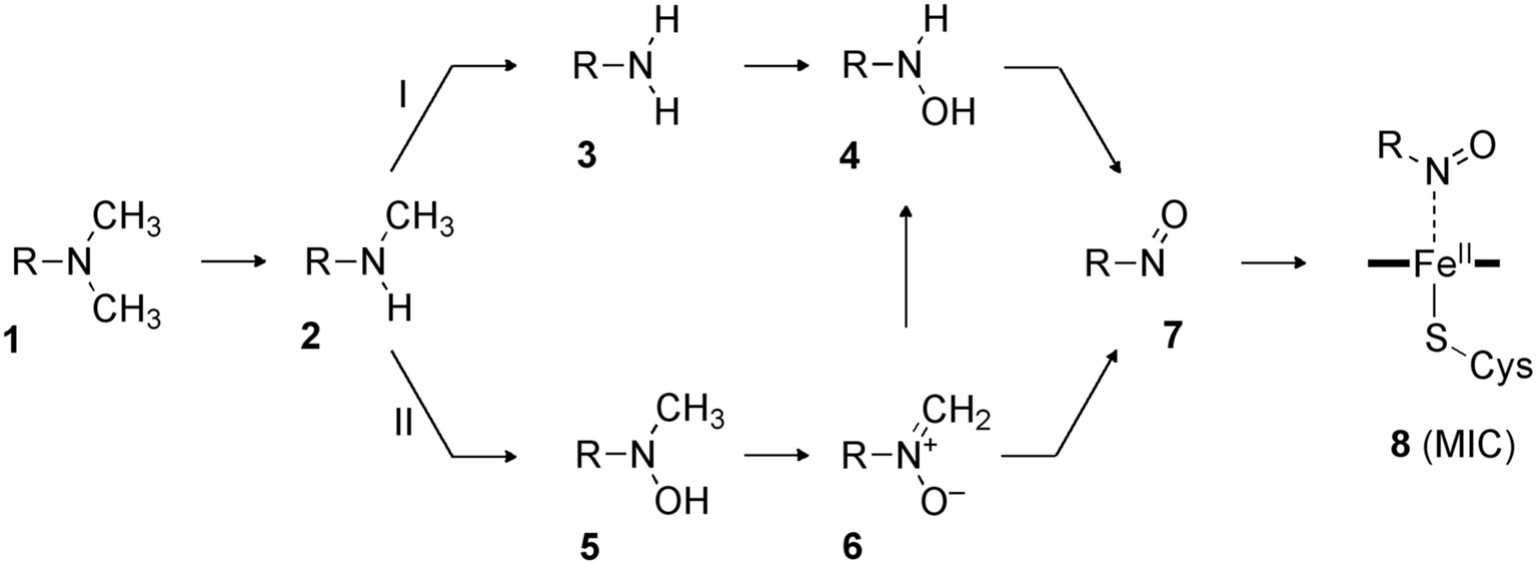
**Likely pathways of MIC formation starting from a tertiary amine (Hanson et al., 2010).** Adapted from Hirao et al. (2013b).

**Figure 6:**
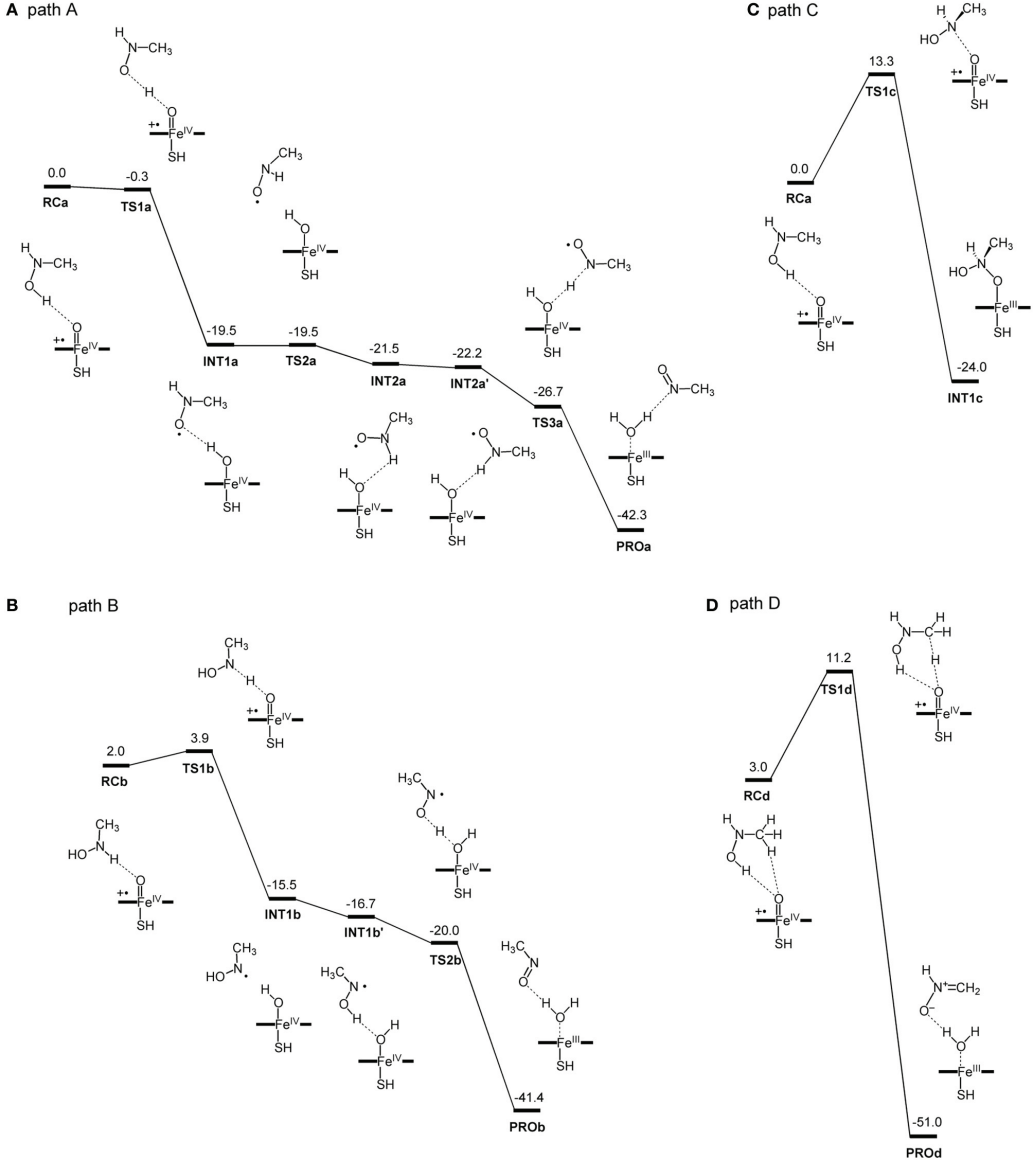
**DFT-calculated reaction energy profiles (in kcal/mol) for paths **(A–D)**, which are initiated by H-abstraction from the O–H bond **(A)**, H-abstraction from the N–H bond **(B)**, N-oxidation **(C)**, and H-abstraction from the methyl group **(D)**.** Reprinted from Hirao et al. (2013b).

**Figure 7:**
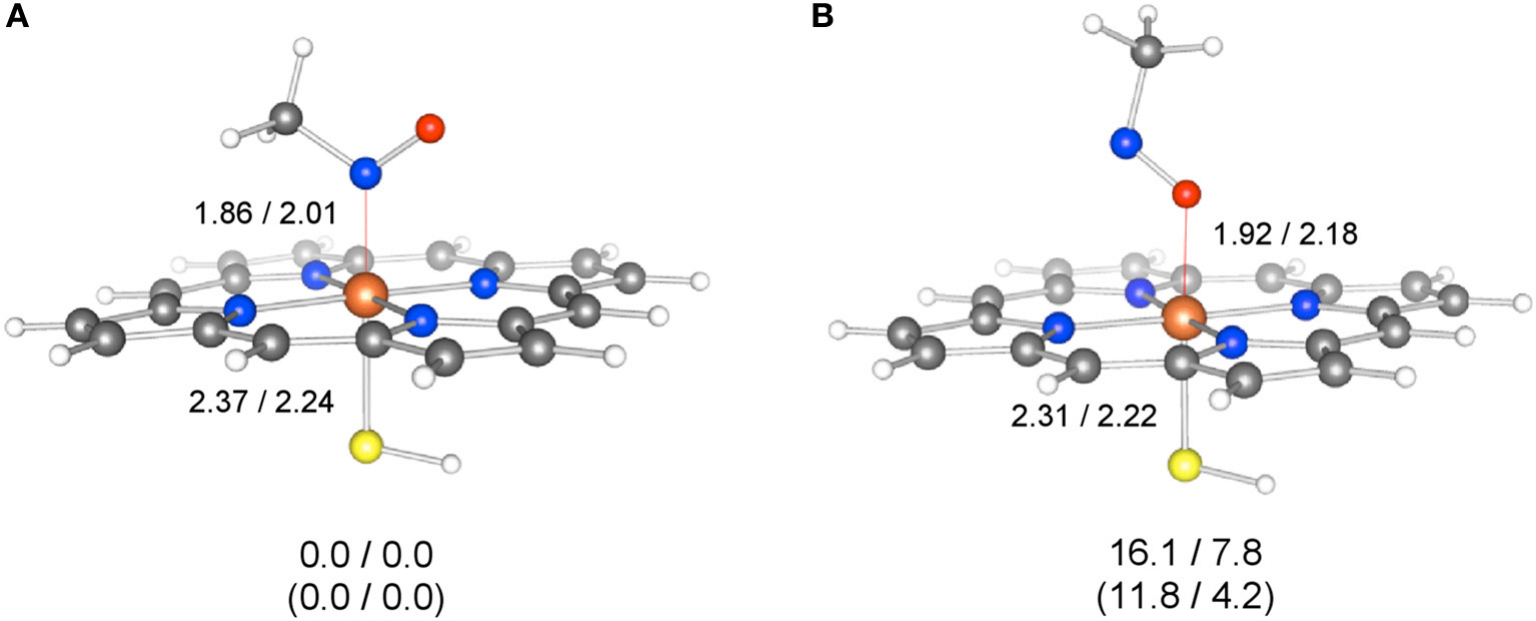
Geometries of N-bound (A) and O-bound (B) forms of ^1^MIC(II) and ^2^MIC(III), optimized at the M06/[SDD(Fe),6-31G^*^(other)] level. Key distances are given in Å. The values below the geometries are relative energies (kcal/mol) obtained at the M06(SCRF)/6-311+G(d,p) level (^1^MIC(II)/^2^MIC(III)), while the values in parentheses are relative energies obtained at the B3LYP(SCRF)/6-311+G(d,p) level. Reprinted from Hirao et al. (2013b).

More recently, Hirao et al. investigated part of the MBI process caused by amine-containing drugs (Hirao et al., 2013b). Amines are prone to form nitrosoalkane metabolic intermediates in P450-mediated metabolism, and the nitrosoalkanes form a quasi-irreversible metabolic-intermediate complex (MIC) to inhibit P450s (Scheme 6) (Hanson et al., 2010). In fact, many existing drugs contain an amine moiety, and thus this metabolic pathway presents a serious concern. The study conducted by Hirao et al. focused on the step from **4** to **7** in Scheme 6, and their comparative DFT study showed that the mechanisms involving H-abstraction from the O–H bond or from the N–H bond of **4** had particularly low energy barriers (paths A and B in Figure 6), suggesting that the reaction proceeds via either of these mechanisms. The mechanisms involving N-oxidation and H-abstraction from a C–H bond had much higher barriers. The coordination bonding in the MIC was also studied in detail. The N-bound form of a MIC was more stable than the O-bound form (Figure 7). Also, the ferrous MIC (^1^MIC(II)) had somewhat larger binding energy than the ferric MIC (^2^MIC(III)). This result was consistent with the fact that a ferrous MIC is formed in the MBI of amine-containing compounds.

#### Analyses of protein environmental effects

Another important aspect of P450s is the protein environmental effect on their active-site properties. P450s accommodate a heme cofactor and organic substrates in their active sites, thereby catalyzing chemically difficult reactions. There have been several QM/MM studies of P450s (Schöneboom et al., 2002, 2004, 2005; Guallar and Friesner, 2004; Schöneboom and Thiel, 2004; Lin et al., 2004; Altun and Thiel, 2005; Bathelt et al., 2005, 2008; Altun et al., 2006, 2007, 2008; Cohen et al., 2006; Harvey et al., 2006; Zheng et al., 2006; Fishelovitch et al., 2007; Cho et al., 2008, 2011b; Hirao et al., 2008c; Wang et al., 2008, 2009b; Porro et al., 2009; Tian and Friesner, 2009; Lai and Shaik, 2011; Lai et al., 2011; Schyman et al., 2011; Lonsdale et al., 2011, 2013; Krámos et al., 2012; Kwiecień et al., 2012; Usharani et al., 2012; Dumas et al., 2013; Li and Shaik, 2013; Li et al., 2013; van der Kamp and Mulholland, 2013; Krámos and Oláh, 2014), which demonstrated that the effect of the protein surroundings is often very important. To gain fundamental insights into the interaction between the active site and the surrounding atoms of P450s, Hirao and co-workers have performed energy decomposition analysis (EDA) studies (Hirao, 2011a; Thellamurege and Hirao, 2013, 2014).

**Figure 8:**
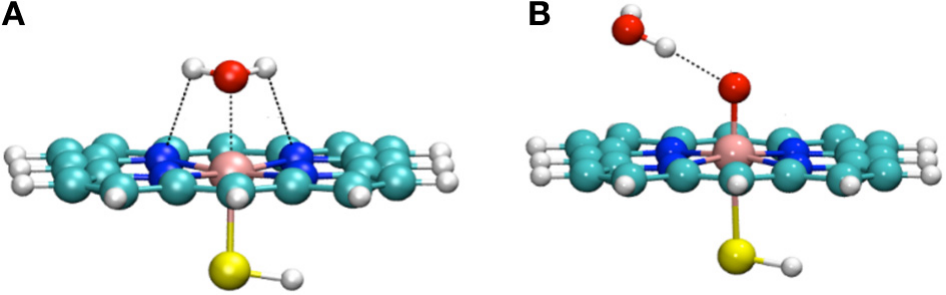
**(A)** The resting state of the P450 catalytic cycle **(B)** Cpd I interacting with a water molecule. Reprinted from Thellamurege and Hirao (2013).

Thellamurege and Hirao performed an DFT-based EDA study of the interaction between two key intermediates of P450 and a molecule of water (Figure 8) (Thellamurege and Hirao, 2013). They used two different types of EDA: the localized MO energy decomposition analysis (LMOEDA), which was developed by Su et al. and is implemented in GAMESS software (Schmidt et al., 1993; Gordon and Schmidt, 2005; Su and Li, 2009), and the EDA method, which is implemented in the Amsterdam Density Functional (ADF) program (Ziegler and Rauk, 1979a,b; ADF2012.01, 2012). In the LMOEDA, the total interaction energy is decomposed into electrostatic, exchange, repulsion, polarization, and dispersion components, whereas in the ADF-EDA, the total interaction energy is decomposed into electrostatic, Pauli repulsion, and orbital interaction terms. Such decomposition analyses provide chemically valuable insights (Kitaura and Morokuma, 1976; von Hopffgarten and Frenking, 2012). The LMOEDA and ADF-EDA were applied to two types of P450–H_2_O complexes. One is the resting state of P450, in which the water molecule is bound to the Fe(III) ion (Figure 8A). In the other complex, the oxo ligand of Cpd I forms a hydrogen bond with a water molecule (Figure 8B). The latter interaction had been suggested to reduce the activation barrier for the H-abstraction by P450 Cpd I (Altun et al., 2006; Kumar et al., 2011). Our EDA study revealed that the main driving force of the interaction in the resting state is the electrostatic energy. The polarization and exchange energies also play significant roles, but to a lesser extent. Compared with this metal–ligand interaction in the resting state, the interaction between Cpd I and H_2_O is weaker because it is viewed as a hydrogen bond. We observed that the electrostatic interaction plays a significant role here. Interestingly, polarization also contributes to a similar extent. This was different from the hydrogen bonding in the water dimer, where a larger contribution to the attractive interaction comes from the electrostatic term.

**Figure 9:**
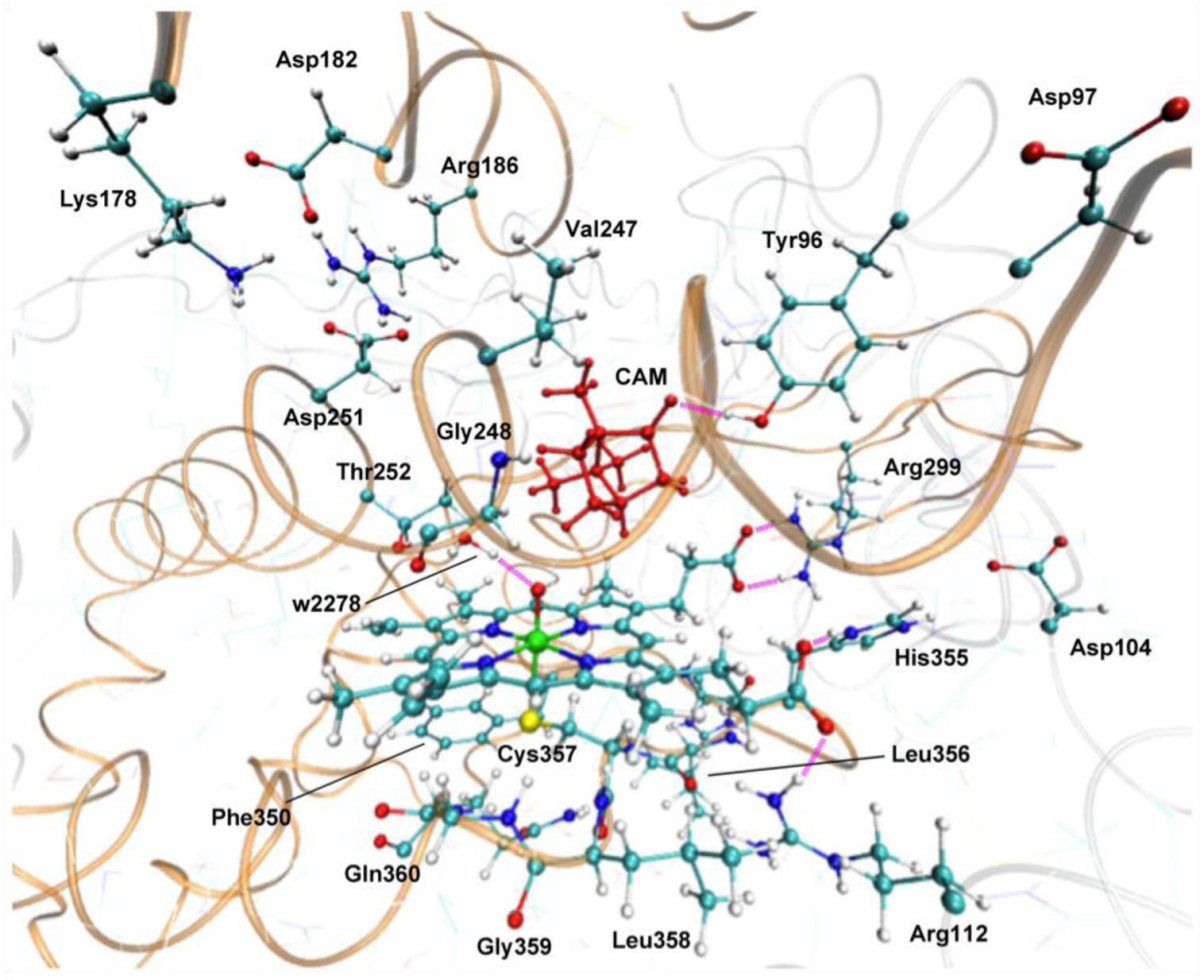
**Active site of P450cam Cpd I.** Reprinted from Hirao (2011a) with permission from the Chemical Society of Japan.

**Figure 10:**
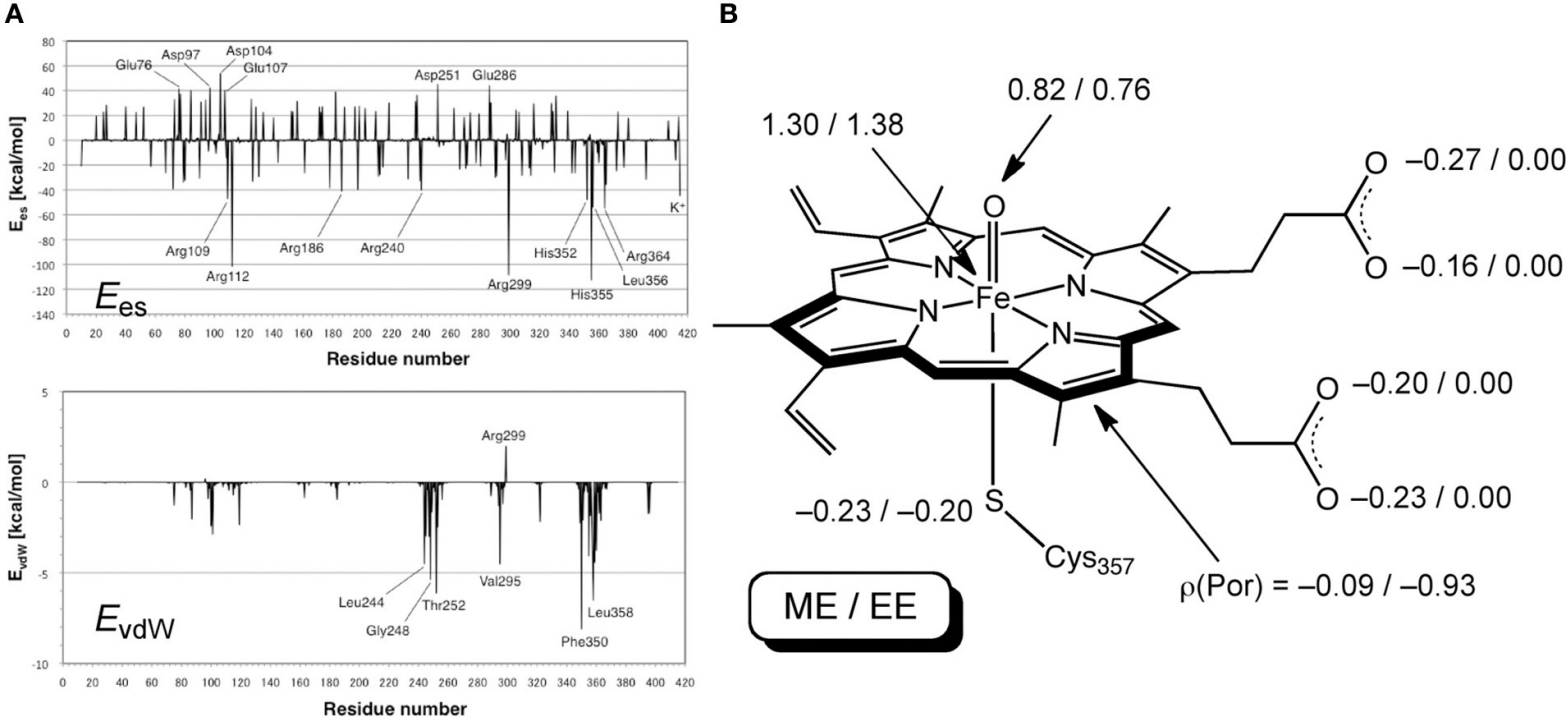
**(A)** *E*_es_ and *E*_vdW_ of each residue. **(B)** Key atomic spin populations calculated by ONIOM-ME and ONIOM-EE calculations. Adapted from Hirao (2011a) with permission from the Chemical Society of Japan.

Hirao performed an EDA of the protein environment effect within bacterial cytochrome P450cam Cpd I (Figure 9), using the ME and EE schemes of ONIOM(DFT:MM) (Hirao, 2011a). B3LYP/[SDD(Fe),6-31G^*^(others)] and AMBER94 were used as the QM and MM methods, respectively. The QM-MM non-bonding interaction energy was decomposed into electrostatic, van der Waals (vdW), and QM polarization terms. The EDA study demonstrated the particular importance of the electrostatic effect. The other effects were also large, but were less significant than the electrostatic effect. The electrostatic and vdW interaction energies were further decomposed into contributions from individual residues (Figure 10A). Positively charged nearby residues, especially those interacting with the two propionate groups of the heme (Arg112, Arg299, and His355), were shown to play particularly important roles in electrostatically stabilizing the active site. The electrostatic stabilization caused by each of these three residues was >100 kcal/mol. The protein environment also affects the spin distribution in the active site (Figure 10B). In the ONIOM-ME calculation, the protein electrostatic effect on the QM electronic state is not taken into account. With this method, significant amounts of spin populations were unnaturally localized on the propionate oxygen atoms. However, with the ONIOM-EE scheme, the unpaired electron shifted to the porphyrin ring.

**Figure 11:**
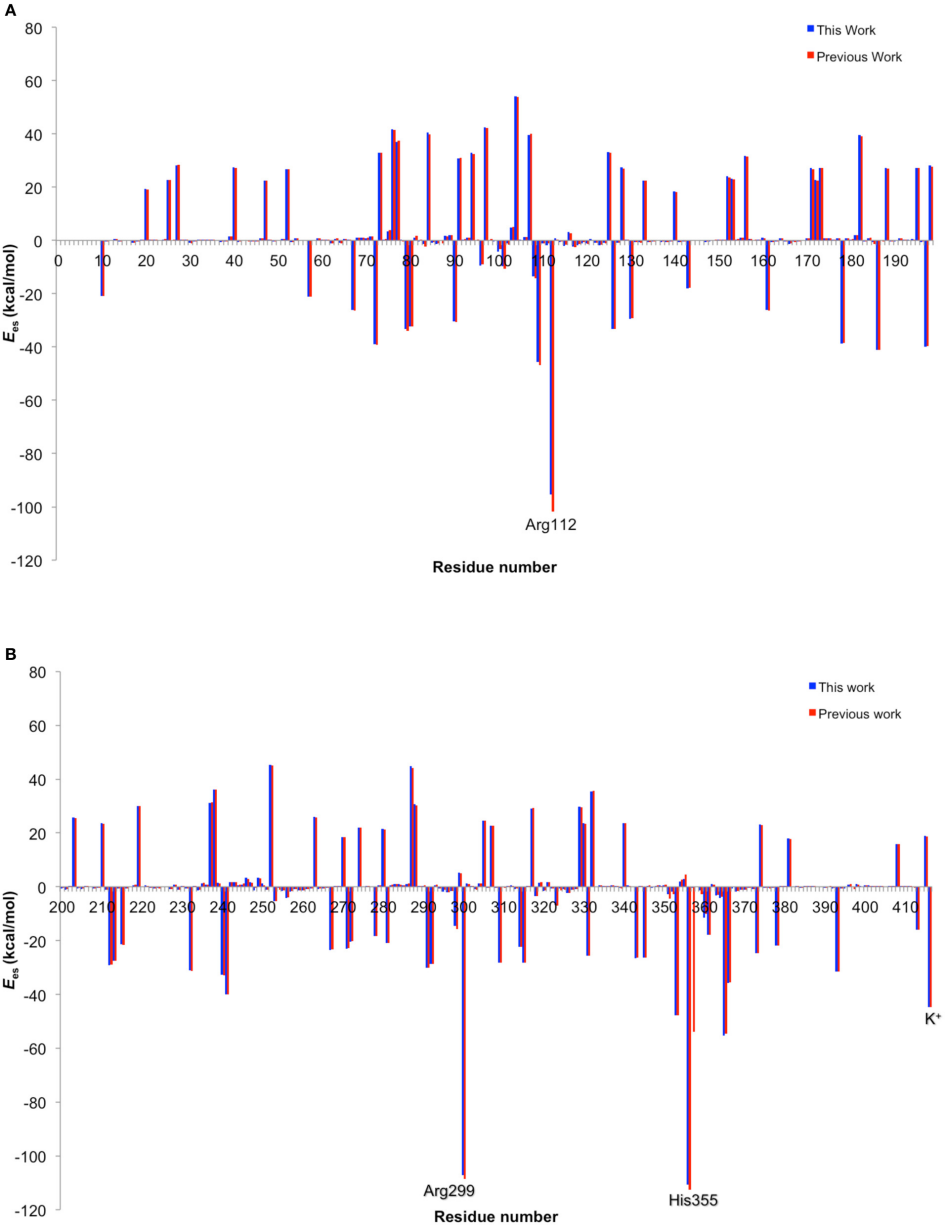
**Comparison of electrostatic energy contributions from amino acid residues 10-199 (A) and 200-414 and K^+^ (#415) (B), obtained in this work with method 1 (blue) and in a previous work (red).** Reprinted from Thellamurege and Hirao (2014) with permission from the American Chemical Society.

**Figure 12:**
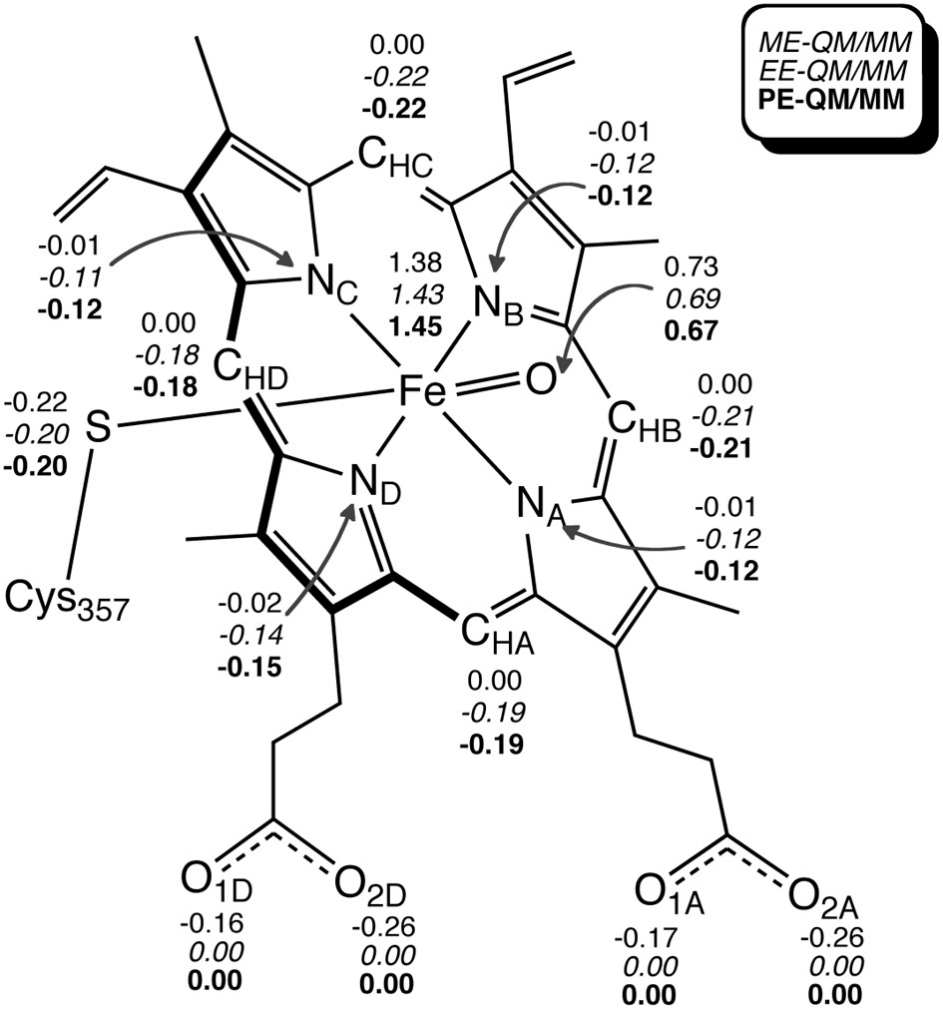
**Key Mulliken atomic spin populations obtained with ME-QM/MM (gas-phase), EE-QM/MM, and PE-QM/MM.** Reprinted from Thellamurege and Hirao (2014) with permission from the American Chemical Society.

The EE scheme of QM/MM is often used because of its capability to describe the polarization of QM atoms in response to the atomic charges of surrounding atoms. However, in reality, the surrounding atoms will also undergo polarization. Such polarization can be described by a PE-QM/MM method. Thellamurege and Hirao performed ME-, EE-, and PE-QM/MM calculations for P450cam (Thellamurege and Hirao, 2014). An additive QM/MM method implemented in GAMESS was used, and the B3LYP/6-31G^*^ method and the AMBER99/QP302 force fields (Thellamurege et al., 2013) were used for the QM and MM subsystems, respectively. This study again showed that the electrostatic interaction stabilizes the QM atoms most significantly. It was shown that when no charge adjustment was made for MM atoms at the QM-MM boundary, the MM point charges caused overpolarization of the QM density. Eliminating the charges of at least the MM atoms at the QM-MM boundary was deemed necessary to avoid overpolarization. Although the method of calculating the electrostatic interaction energy as well as the QM and MM methods used here were slightly different from those used in Hirao (2011a), the two studies yielded very similar residue-resolved electrostatic interaction energies (Figure 11). The MM polarization effect that was described by PE-QM/MM did not change the spin distribution within the QM atoms significantly (Figure 12). The dipole moments calculated by EE-QM/MM and PE-QM/MM were also very similar.

### Non-heme iron enzymes

#### Myo-inositol monooxygenase

**Scheme 7:**
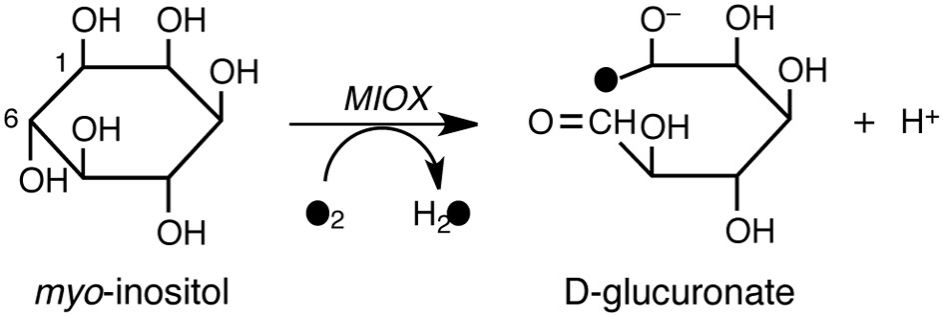
**MIOX-catalyzed conversion of *myo*-inositol into D-glucuronate.** Reprinted from Hirao and Morokuma (2009) with permission from the American Chemical Society.

*myo*-Inositol monooxygenase (MIOX) is a non-heme diiron enzyme that is presumed to be involved in the pathogenesis of diabetic complications (Xing et al., 2006; Bollinger et al., 2009). MIOX catalyzes the first committed step in *myo*-Inositol catabolism, namely, the conversion of *myo*-inositol into D-glucuronate (Scheme 7). This conversion is initiated by H-abstraction from the C1 atom of *myo*-inositol by a ferric superoxide moiety. The reaction mechanism was investigated by Hirao and Morokuma using DFT and ONIOM(DFT:MM) calculations (Hirao and Morokuma, 2009). The calculations predicted that the barrier for the O–O bond cleavage is higher than that for the H-abstraction step. This explained why the experimentally determined kinetic isotope effect on the steady state turnover was close to unity.

**Scheme 8:**
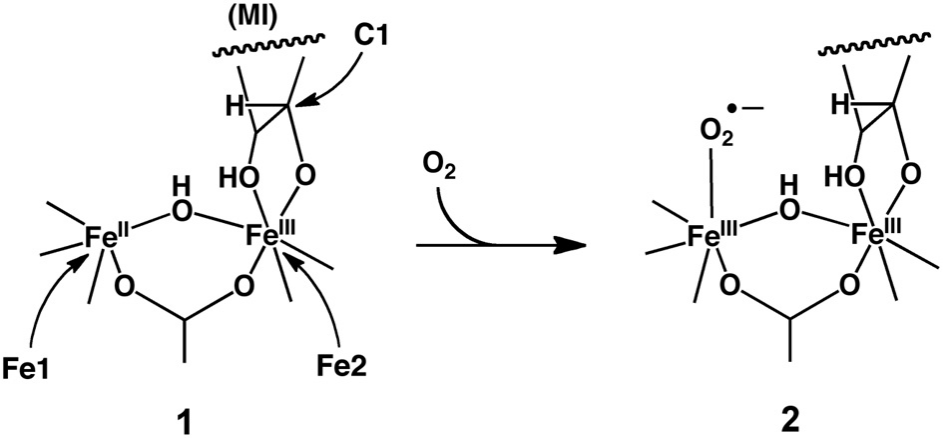
**O_2_ Binding in MIOX to form a ferric-superoxide species.** Reprinted from Hirao (2011b) with permission from the American Chemical Society.

Hirao further performed an ONIOM-based EDA study of MIOX before (**1**) and after (**2**) the O_2_ binding to the iron (Scheme 8) (Hirao, 2011b). The ONIOM-EE calculation yielded an O_2_ binding energy of 3.5 kcal/mol, which might be somewhat underestimated (Siegbahn, 2006). For both intermediates, the electrostatic stabilization energy was the largest of all the decomposed energy terms. However, the difference in electrostatic stabilization between **1** and **2** was very small, indicating that the electrostatic protein effect may not make a significant contribution to the ferric-superoxide formation. Rather, the empirically estimated dispersion effect had a relatively large stabilizing effect on the O_2_ binding process.

#### 2-hydroxylethylphosphonate dioxygenase

**Scheme 9:**
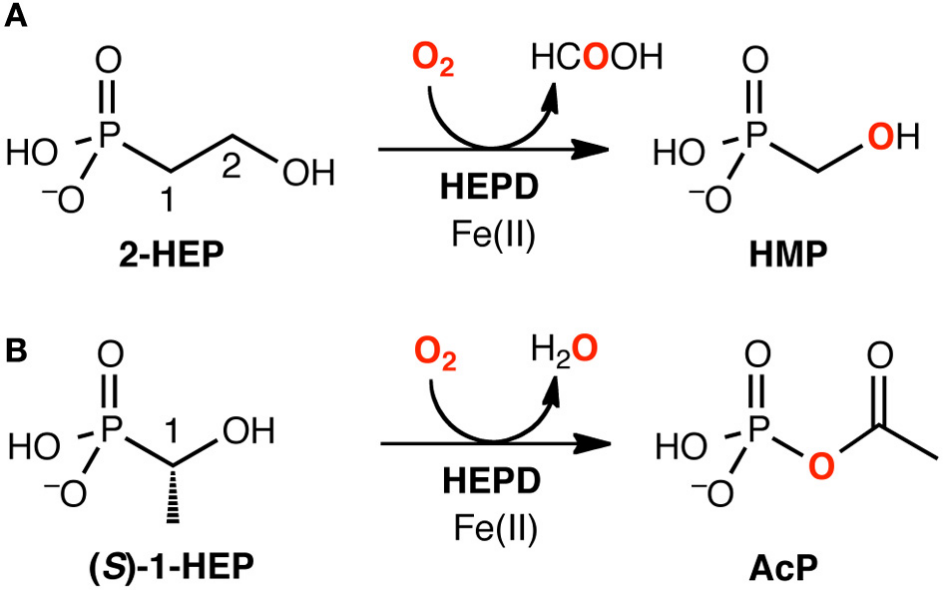
**HEPD-catalyzed reactions of **(A)** 2-HEP and **(B)** 1-HEP.** Reprinted from Hirao and Morokuma (2011b) with permission from the American Chemical Society.

**Scheme 10:**
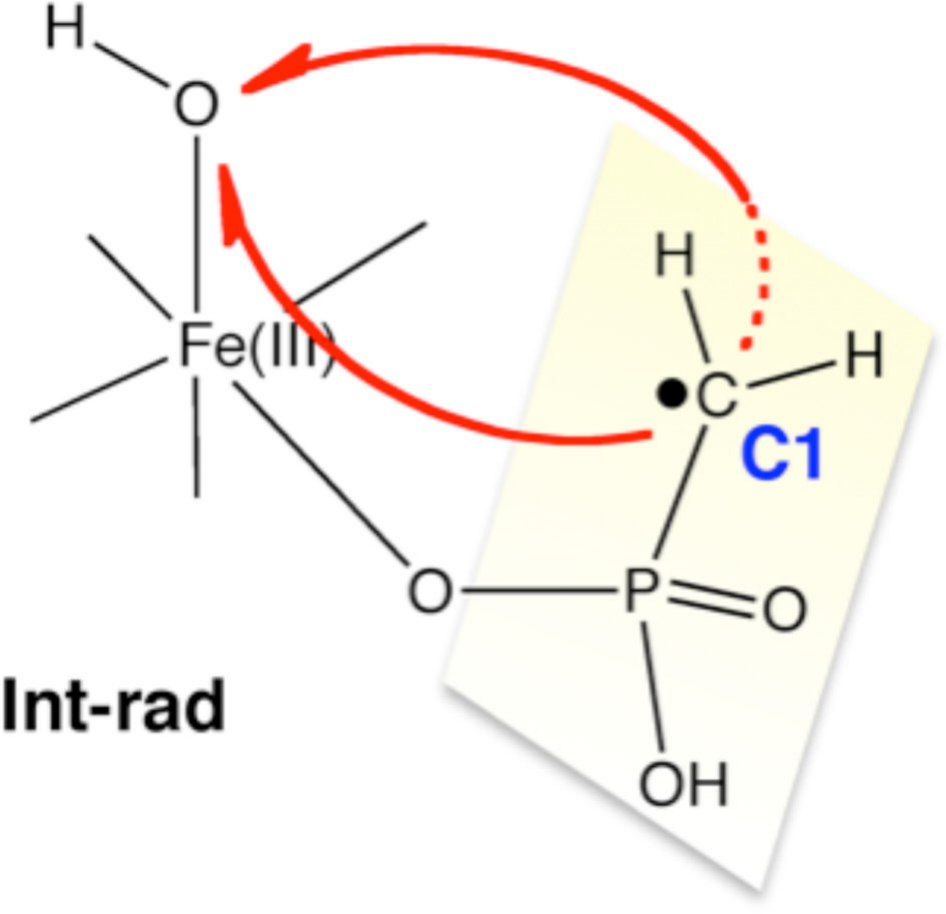
**Theoretically proposed radical intermediate.** Reprinted from Hirao and Morokuma (2011b) with permission from the American Chemical Society.

**Figure 13:**
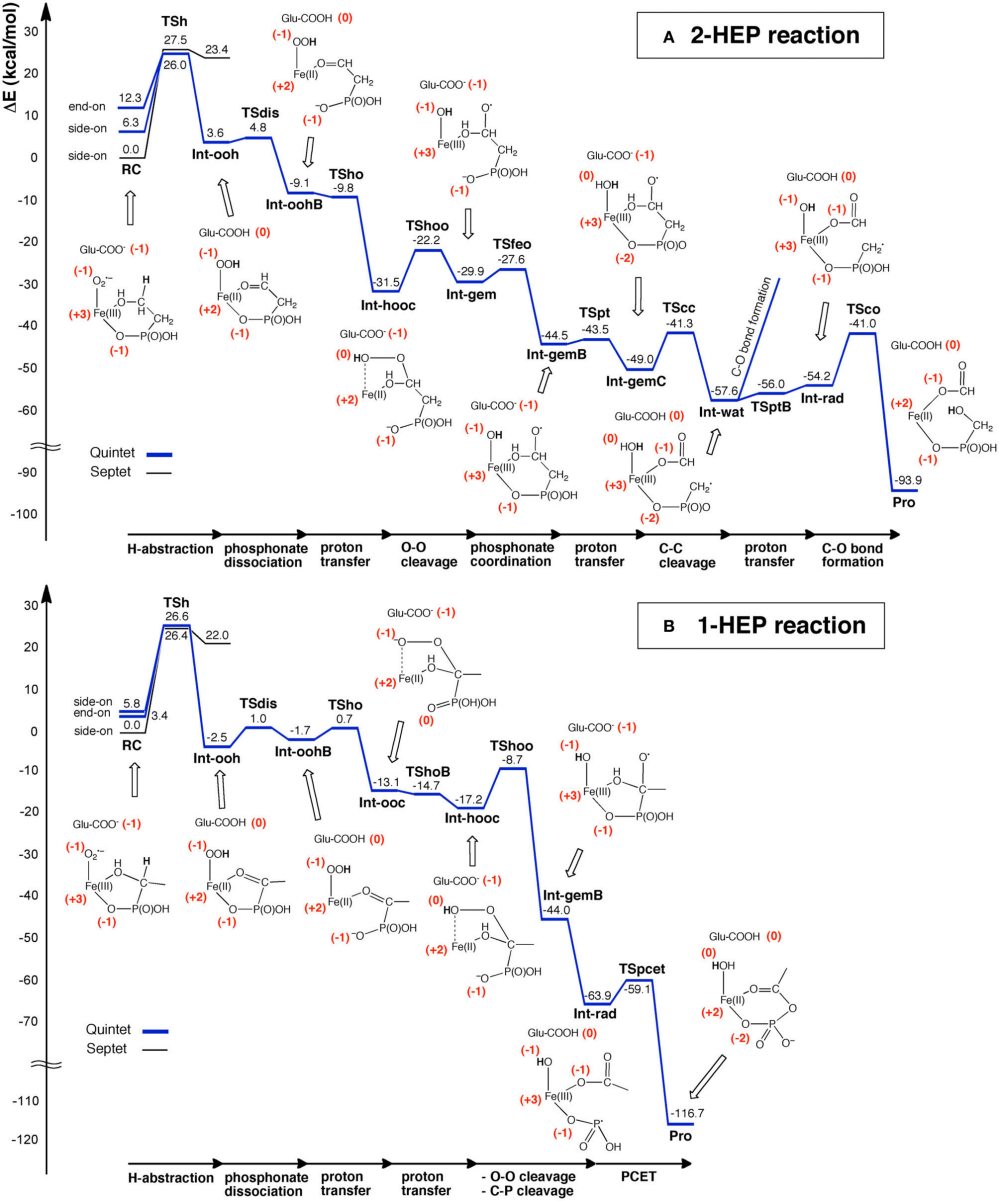
**Energy profiles for the reactions of **(A)** 2-HEP and **(B)** 1-HEP, determined by ONIOM(DFT:MM) calculations.** Reprinted from Hirao and Morokuma (2011b) with permission from the American Chemical Society.

Using DFT and ONIOM(DFT:MM) calculations, Hirao and Morokuma investigated the enzymatic reactions of a non-heme iron enzyme, 2-hydroxylethylphosphonate dioxygenase (HEPD) (Scheme 9) (Cicchillo et al., 2009; Whitteck et al., 2009, 2011; Hirao and Morokuma, 2010a, 2011b; Peck et al., 2011). Their DFT study of the HEPD-catalyzed reaction of 2-hydroxylethylphosphonate (2-HEP) substrate suggested that a radical intermediate should be formed in the late stage of the reaction (Hirao and Morokuma, 2010a). Such a radical intermediate was again observed in their ONIOM study (**Int-rad** in Scheme 10) (Hirao and Morokuma, 2011b). According to this scenario, the radical intermediate should have a sufficiently long lifetime to allow it to rotate about the P–C bond. As such, the radical should be able to attack the Fe^III^–OH from either face of the P–CH_2_• moiety. Whitteck et al. performed stereochemical experiments in which they introduced a deuterium atom into C1 of 2-HEP. Interestingly, the stereochemistry at C1 was lost in the hydroxymethylphosphonate product (Whitteck et al., 2011), which is consistent with our theoretically predicted radical-involving mechanism. The reaction energy profile for 1-HEP was mostly similar to that for 2-HEP (Figure 13), especially in the early phase of the reaction (Hirao and Morokuma, 2011b). However, at a late stage of the reaction, proton-coupled electron transfer was observed: after a homolytic P–C bond cleavage, an unpaired electron on the phosphorous transferred to the iron center to create an electron-deficient site, while there was proton transfer from the phosphate moiety to the hydroxyl group of ferric hydroxide. The formation of an electron-deficient site triggered the attack of the oxygen of acetate on the phosphorus, and P–O bond formation was accomplished.

### Other challenges

Although the usefulness of DFT calculations in studying iron-containing molecules has been well documented, there is a long way to go before DFT acquires the capability to describe iron-containing systems or any other transition-metal-containing systems perfectly (Harvey, 2006; Cramer and Truhlar, 2009).

First, reliable exchange-correlation functionals will need to be developed to describe accurately the spin-state ordering in various species. This goal is particularly important for studies of iron-containing molecules because multiple spin states often coexist within a narrow energy range. It is still not entirely clear which existing functional is most universally accurate for the description of iron-containing molecules in different oxidation states. For calibration purposes, comparisons of DFT with wave-function theories are often useful (Radoń and Broclawik, 2007; Swart, 2008; Chen et al., 2010, 2012; Vancoillie et al., 2010). Swart computed spin-state energies of several iron complexes that have an oxidation state of +2, +3, or +6, using CASPT2 and DFT methods, and suggested that the OPBE functional gives good results (Swart, 2008). Chen et al. applied CASPT2/MM to P450 and chloroperoxidase Cpd I, and showed that B3LYP/MM performed very well (Chen et al., 2010). Chen et al. also assessed the reliability of the B3LYP functional in calculating the spin-state ordering of the [(TMC)Fe(O_2_)]^2+^ species. To this end, they truncated the system to a smaller model ([(NH_3_)_4_Fe(O_2_)]^2+^) and applied the B3LYP and RCCSD(T) methods to the model. Their calculations showed that for most of the states, the relative energies obtained from these two methods were more or less similar (Chen et al., 2012).

In general, accurate evaluation of barrier heights of chemical reactions is a particularly important goal of DFT calculations, because barrier heights are directly linked to rate constants (Claeyssens et al., 2006; Llano and Gauld, 2010; Cho et al., 2012b). In many cases, DFT and DFT/MM provide reasonable trends in barrier heights at least qualitatively and are thus highly useful for mechanistic investigations. However, theoretically determined free energy barriers of reactions are often quite different from experimental ones. The reaction rate constant depends exponentially on the free energy barrier (according to the Eyring equation), and thus agreement of the rate constant between theory and experiment is even worse than that for the free energy. One of the major reasons for the error must be the insufficient accuracy of DFT functionals. Thus, DFT may not always be able to estimate reaction barriers accurately for a given spin state. In addition, the above-mentioned potentially poor descriptions of spin-state orderings could affect the accuracy of barrier heights, because there may be a switch in the spin state on a reaction pathway for iron-containing molecules. Reaction barriers are also influenced significantly by many weak interactions outside the reaction center, and in this respect, the dispersion effect is an important factor that has to be taken into account when improving DFT (Lynch and Truhlar, 2001; Zhao et al., 2004, 2005; Zhao and Truhlar, 2004; Grimme, 2006; Karton et al., 2008; Lonsdale et al., 2010, 2012; Siegbahn et al., 2010). Aside from the inherent problems of DFT, there are many other sources that make calculated free energy barriers of enzymatic reactions inaccurate (Llano and Gauld, 2010). For example, the effects of entropy (Hu and Yang, 2008; Lundberg et al., 2009) and tunneling (Cui and Karplus, 2002; Knapp and Klinman, 2002; Knapp et al., 2002; Hatcher et al., 2004; Olsson et al., 2004a,b; Tejero et al., 2006; Hammes-Schiffer and Soudackov, 2008; Hammes-Schiffer et al., 2008; Iyengar et al., 2008; Klinman, 2009; Phatak et al., 2012; Hammes-Schiffer, 2013) affect the rate constants of enzymatic reactions (or even synthetic complexes) profoundly. For realistic descriptions of enzymatic reactions, the reaction dynamics of iron enzymes may also be investigated actively using QM/MM (Lian et al., 2013).

The chemical reactions of iron enzymes often appear to be very simple. For example, a reaction may involve only the transfer of a hydrogen atom; however, the nature of the hydrogen transfer could differ critically. In some cases hydrogen may undergo “hydrogen atom transfer (HAT),” while in other cases, it may undergo “proton-coupled electron transfer (PCET).” Thermodynamic parameters such as redox potentials and p*K*_a_-values provide valuable insights into these ubiquitous processes; thus, theoretical evaluation of these quantities using DFT or DFT/MM is a key challenge (Kamerlin et al., 2009; Siegbahn and Blomberg, 2010; Hughes and Friesner, 2012; Castro and Bühl, 2014). Apart from the quantitative treatment of hydrogen-transfer processes, devising ways to derive chemical insight into the mechanistic complexities underlying these processes also presents an important goal (Shaik et al., 2010b,c; Usharani et al., 2013a,b).

The reactivity patterns of iron-containing enzymes or complexes cannot be easily generalized. For example, non-heme iron enzymes use not only iron(IV)-oxo or ferric-superoxide reactive species (Ye and Neese, 2009; Lundberg and Borowski, 2013), but also iron(III)-hydroxide to trigger a reaction (Knapp and Klinman, 2002; Knapp et al., 2002; Hatcher et al., 2004; Olsson et al., 2004a,b; Tejero et al., 2006; Hammes-Schiffer et al., 2008; Hammes-Schiffer and Soudackov, 2008; Iyengar et al., 2008; Klinman, 2009; Hirao and Morokuma, 2010b; Phatak et al., 2012; Hammes-Schiffer, 2013). DFT and DFT/MM investigations of intermediates and reactions of individual enzymes and synthetic complexes will continue to play valuable roles in their characterization. DFT-based spectroscopic studies of reaction intermediates will also provide us with crucial information on iron enzymes and complexes (Neese, 2006, 2009; Orio et al., 2009; Römelt et al., 2009; Chandrasekaran et al., 2011).

## Summary

We have reviewed several recent applications of DFT to iron-containing synthetic complexes and enzymes, but not exhaustively. DFT provides insight into structural and spectroscopic features, chemical reaction mechanisms, etc., often with high reliability. Furthermore, DFT can be combined with other theoretical techniques, such as EDA schemes, to gain deeper insight into the nature of molecular interactions. Even for systems that contain thousands of atoms, we can still apply DFT by employing some hybrid approach (e.g., DFT/MM or DFT/DFT). Clearly, DFT is already a useful tool for the investigation of fundamental aspects of molecules. We believe that, in the future, DFT will become more accurate and play increasingly important roles, especially in practical areas such as catalyst design and drug design.

### Conflict of interest statement

The authors declare that the research was conducted in the absence of any commercial or financial relationships that could be construed as a potential conflict of interest.
